# Low arterial oxygen-content burden during the first 24 h and hospital mortality in mechanically ventilated adults: analyses of MIMIC-IV and eICU

**DOI:** 10.3389/fmed.2026.1935082

**Published:** 2026-10-09

**Authors:** Xiaoyan Gao, Jiaqi Wang, Luyan Xiong, Bingzhu Hu, Cheng Jiang, Juan Li, Shi Chen, Cong Zhang

**Affiliations:** 1 Department of Pulmonary and Critical Care Medicine, The Sixth Hospital of Wuhan, Affiliated Hospital of Jianghan University, Wuhan, Hubei, China; 2 Hubei Key Laboratory of Cognitive and Affective Disorders, School of Medicine, Jianghan University, Wuhan, Hubei, China; 3 Community Health Service Center of Minquan Street, Jianghan District, Wuhan, Hubei, China

**Keywords:** arterial oxygen content, critical care, eICU, hemoglobin, mechanical ventilation, MIMIC-IV, replication, time-weighted burden

## Abstract

**Background:**

Arterial oxygen content (CaO_2_) combines hemoglobin-bound and dissolved oxygen. A time-weighted CaO_2_ deficit could capture changing anemia and desaturation that a hemoglobin-only summary misses. We examined its association with hospital mortality and its added value beyond hemoglobin.

**Methods:**

In this retrospective cohort study, we included mechanically ventilated adults from the Medical Information Mart for Intensive Care IV (MIMIC-IV) and eICU Collaborative Research Database (eICU). The MIMIC-IV 25th percentile of the first matched CaO_2_ value (12.48 mL O_2_/dL) was prespecified as an empirical cutoff and was applied unchanged to eICU. The first-24-h burden integrated the time-weighted deficit below this cutoff. Models were adjusted for demographics and diagnosis-derived case mix. We compared CaO_2_ burden with hemoglobin burden calculated at the same blood-gas time points and performed landmark, measurement-coverage, severity, and *post hoc* peripheral oxygen saturation (SpO_2_)-stratified analyses.

**Results:**

Burden was available for 14,050 MIMIC-IV and 20,780 eICU stays. CaO_2_ was strongly correlated with hemoglobin (*r* = 0.965 and 0.949, respectively), which explained *R*^2^ = 0.932 and 0.901 of first-measurement CaO_2_ variation, respectively; median dissolved-oxygen contributions were 2.9% and 2.5%, respectively. Per 1-SD higher burden, adjusted odds ratios for hospital mortality were 1.33 (95% confidence interval 1.28–1.39) in MIMIC-IV and 1.29 (1.24–1.35) in eICU. Additional Acute Physiology and Chronic Health Evaluation adjustment attenuated the eICU estimate to 1.14 (1.10–1.19), while 24-h landmark estimates were 1.26 (1.21–1.32) and 1.19 (1.14–1.25), respectively. Cross-validated area under the receiver operating characteristic curve (AUROC) gains over matched hemoglobin burden were 0.006 and 0.010, respectively, and calibration was similar. In *post hoc* analyses, hemoglobin explained less CaO_2_ variation when first SpO_2_ was <90% (*R*^2^ = 0.787 and 0.682, respectively) than when it was ≥95% (*R*^2^ = 0.973 and 0.978, respectively); corresponding AUROC gains were 0.045 and 0.044 versus 0.002 in both databases.

**Conclusion:**

In cohorts dominated by preserved oxygen saturation, low CaO_2_ was predominantly an anemia-related signal, and its association with mortality was substantially attenuated by illness-severity adjustment. The burden is best viewed as a compact observational summary of the depth and duration of low arterial oxygen content, not as an independent measure of tissue oxygenation, a superior prediction model, or a treatment target.

## 1 Introduction

Clinicians commonly use peripheral oxygen saturation (SpO_2_) and arterial oxygen tension (PaO_2_) to guide oxygen therapy in mechanically ventilated patients. These measures describe saturation and partial pressure, but not the total amount of oxygen carried in arterial blood. Arterial oxygen content (CaO_2_) combines hemoglobin-bound oxygen with a smaller dissolved component. It is therefore a measure of blood oxygen-carrying capacity, but it is not the same as systemic oxygen delivery, which also depends on cardiac output, perfusion, diffusion, and tissue extraction (1–3).

Repeated measurements can describe a changing intensive care unit (ICU) course better than a single value. Previous studies have examined time-weighted CaO_2_, time at risk based on SpO_2_/FiO_2_, PaO_2_ exposure, time-varying oxygen intensity, and hyperoxia (4–12). These studies also show that when and how often clinicians obtain blood gases can affect observed associations. Less is known about a simple measure that combines the depth and duration of values below a low CaO_2_ cutoff.

There is no widely used, externally validated CaO_2_ threshold for ICU treatment. We therefore prespecified the lower quartile in the Medical Information Mart for Intensive Care IV (MIMIC-IV) as an empirical threshold and applied it unchanged to the eICU Collaborative Research Database (eICU). This choice was intended to define a stable lower-tail exposure, not a biological boundary or a transfusion trigger. We also examined alternative percentiles and numerical cutoffs. Because SpO_2_ is often near the upper end of its range during mechanical ventilation, we expected most CaO_2_ variation to come from hemoglobin. We nevertheless hypothesized that a time-weighted CaO_2_ deficit could capture the combined and changing effects of anemia and desaturation that a hemoglobin-only summary would miss. A key question was therefore whether the combined measure provided clinically meaningful information beyond anemia itself.

A CaO_2_ value calculated from SpO_2_ also inherits the limitations of pulse oximetry, including rounding, ceiling effects, reduced accuracy during desaturation, and racial and ethnic differences in occult hypoxemia (13–19). Direct arterial oxygen saturation (SaO_2_) was not available often enough for the primary exposure, so we examined SaO_2_-based estimates when possible. These limitations reinforce that an electronic-record CaO_2_ measure is not a direct measure of tissue hypoxia.

We studied mechanically ventilated adults in MIMIC-IV and eICU to answer the following four questions: whether a 24-h low-CaO_2_ burden was associated with hospital mortality; whether low content could occur despite preserved SpO_2_; how strongly the measure was driven by hemoglobin; and whether findings persisted after severity, treatment, landmark, measurement-coverage, and repeated-patient checks. The intended contribution was not to rediscover that anemia lowers oxygen content. It was to test a longitudinal, one-number summary, reproduce the association in a second database, and define where the summary did and did not add information beyond hemoglobin.

## 2 Materials and methods

### 2.1 Study design and data sources

We conducted a retrospective cohort study using MIMIC-IV version 3.1 and eICU version 2.0 (20–23), following Strengthening the Reporting of Observational Studies in Epidemiology (STROBE) recommendations (24). MIMIC-IV was used to select the exposure cutoff. That cutoff and the analysis rules were then applied to eICU as an external-cohort replication. MIMIC-IV contributed the earliest eligible ICU stay per patient. eICU contributed the first ICU unit visit in each hospitalization; the same patient could therefore contribute more than one hospitalization, which we addressed in a sensitivity analysis. The primary analysis estimated the adjusted association between low-CaO_2_ burden during ICU h 0–24 and hospital death among patients for whom the burden could be calculated. It was not designed to estimate the effect of oxygen, transfusion, or another treatment.

### 2.2 Ethics and data access

The study protocol was reviewed by the Ethics Committee of the Affiliated Hospital of Jianghan University, which determined that the present study was exempt from further ethical review because it involved only the secondary analysis of fully deidentified data obtained from credentialed-access research databases, with no direct interaction with participants and no access to identifiable private information. The requirement for obtaining additional informed consent was therefore waived.

The collection and sharing of MIMIC-IV data were reviewed and approved by the Institutional Review Board of Beth Israel Deaconess Medical Center, which granted a waiver of informed consent. The eICU Collaborative Research Database contains data deidentified in accordance with the Health Insurance Portability and Accountability Act Safe Harbor provisions. Access to both databases was granted to credentialed investigators after completion of the required human-subjects research training and execution of the applicable PhysioNet data use agreements. All analyses were conducted in accordance with the relevant institutional requirements and the principles of the Declaration of Helsinki.

### 2.3 Cohort construction and ventilation identification

In MIMIC-IV, invasive ventilation was identified from procedure events item 225792. In eICU, we used treatment and respiratory-charting entries that indicated mechanical ventilation, intubation, a ventilator mode, or active ventilator operation. The upstream cohorts contained 23,811 MIMIC-IV and 38,416 eICU adult invasive-ventilation stays. Eligible stays required at least one PaO_2_ value during ICU h 0–24, a nearest hemoglobin value within ±6 h, a nearest SpO_2_ value within ±2 h, a physiologically valid calculated CaO_2_ value, and known hospital-discharge status. When more than one value was available, the temporally nearest record was selected. These requirements yielded 14,052 MIMIC-IV and 20,802 eICU stays, meaning that 9,759 (41.0%) and 17,614 (45.9%) upstream stays, respectively, did not enter the first-measurement cohort. The available cohort-construction records retained the total number excluded but not mutually exclusive reason-specific counts; therefore, only total exclusions are reported. Repeated-measure burden was unavailable for a further 2 and 22 stays, respectively (Figure 1).

**Figure 1:**
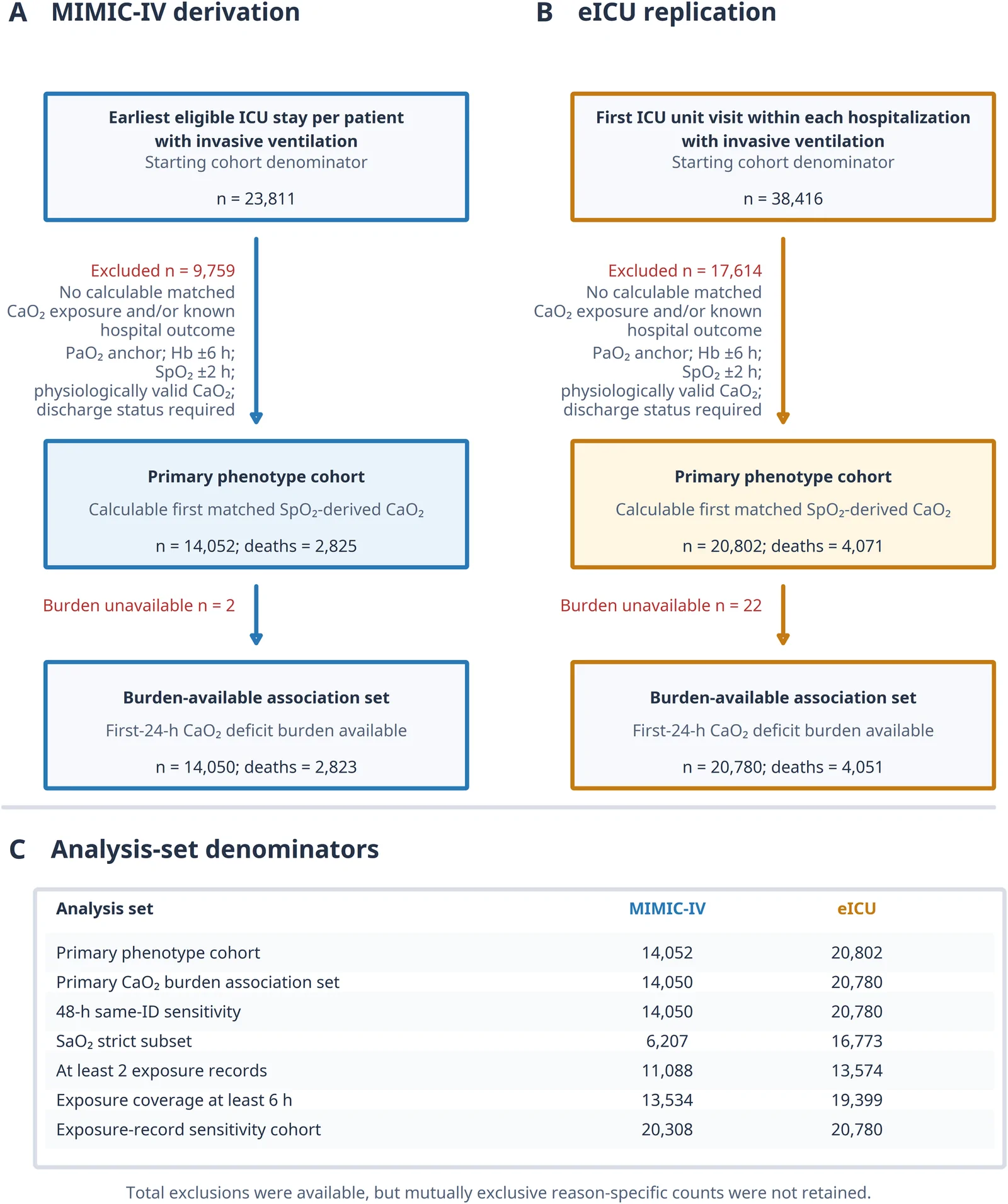
Analytic cohort denominators and two-database study design. **(A)** MIMIC-IV derivation cohort: the earliest eligible ICU stay per patient was used, and MIMIC-IV was used to select the cutoff. **(B)** eICU replication cohort: the first ICU unit visit within each hospitalization was used for external-cohort replication. **(C)** Analysis-set denominators in the two databases. Calculable matched CaO2 exposure and known hospital-discharge status were required. Total exclusions were available, but mutually exclusive reason-specific counts were not retained. Burden was unavailable for 2 MIMIC-IV and 22 eICU stays.

### 2.4 Arterial oxygen content and 24-h burden

At each eligible PaO_2_ time point, CaO_2_ was calculated as 1.34 × Hb × (SpO_2_/100) + 0.003 × PaO_2_, with hemoglobin in g/dL and PaO_2_ in mmHg. In the direct-saturation sensitivity analysis, SaO_2_ within ±0.5 h replaced SpO_2_. The prespecified MIMIC-IV lower quartile of the first matched CaO_2_ measurement was 12.480855 mL O_2_/dL, displayed as 12.48 mL O_2_/dL. This empirical threshold was selected before examining eICU results and was not treated as a clinical intervention threshold. Applying one numerical threshold in both databases created a common replication definition but did not assume that the cutoff occupied the same percentile or had identical meaning under different case mix, sampling frequency, or practice patterns. Deficit at time *t* was max[0, 12.48−CaO_2_(*t*)]. Duplicate blood-gas times were reduced to one record. Each value represented the period until the next eligible record, 6 h after the current record, or ICU hour 24, whichever came first. The 6-h limit was a pragmatic safeguard against carrying an old value too far in irregular electronic-record data; it does not imply that physiology remains unchanged for 6 h. Deficit multiplied by represented time was summed and divided by 24 h. We call this the 24-h low-CaO_2_ burden. Time before the first eligible value or after the 6-h limit was assigned zero deficit, meaning that no unmeasured deficit was imputed. This conservative rule can underestimate burden when unobserved time is truly low, so we examined observed-time and measurement-coverage alternatives (Figure 2).

**Figure 2:**
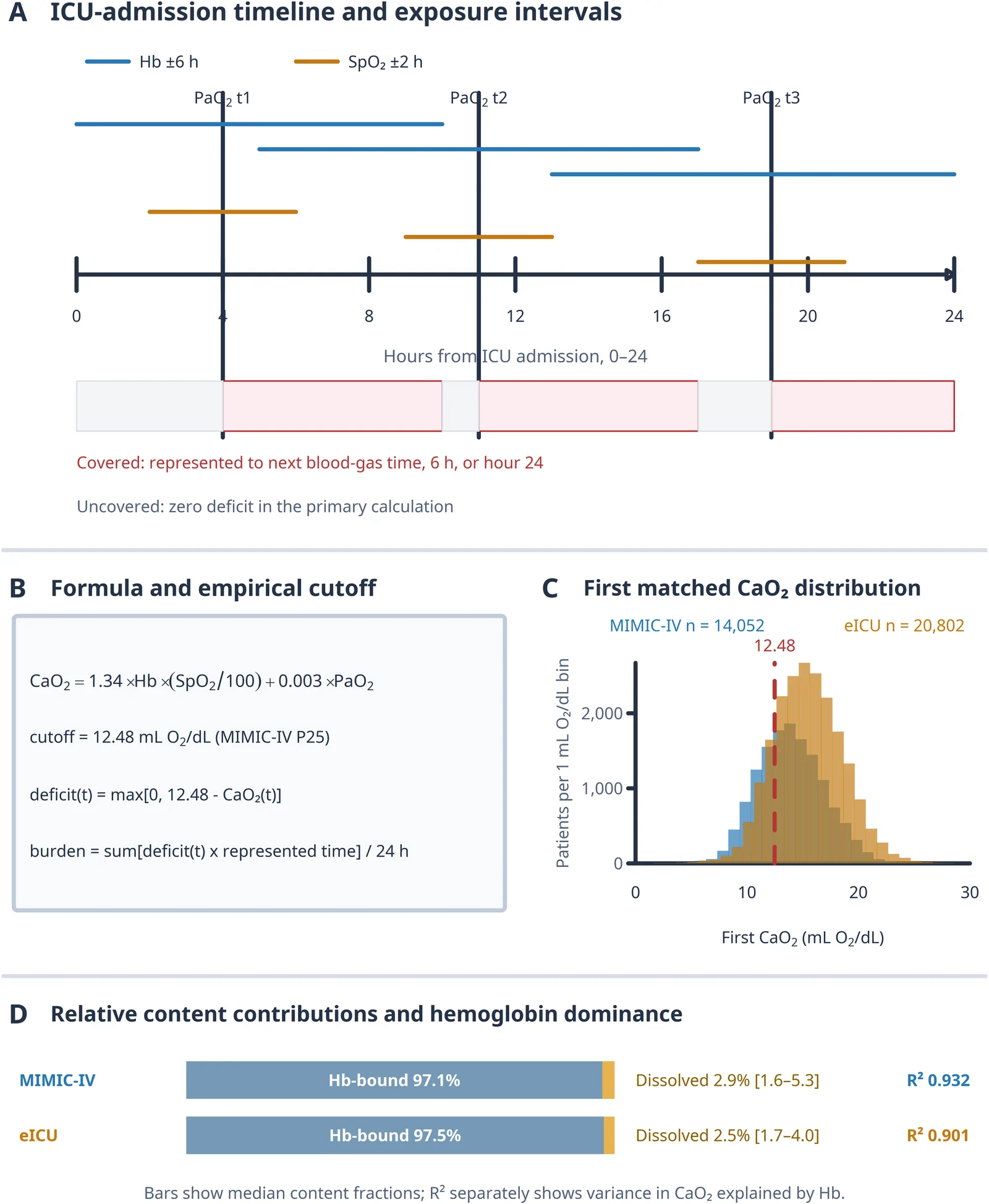
Construction, distribution, and components of the 24-h low-CaO_2_ burden. Panel **(A)** shows PaO_2_ time points, matching windows for hemoglobin and SpO₂, 6-h limits, and covered and uncovered intervals. Panel **(B)** shows the CaO_2_ equation, the empirical MIMIC-IV lower-quartile cutoff, and the deficit calculation. Panel **(C)** shows the distribution of the first matched CaO_2_ value in each database. Panel **(D)** shows the median percentages of CaO_2_ contributed by hemoglobin-bound and dissolved oxygen and separately reports the *R*^2^ from CaO_2_ regressed on hemoglobin. Percent content contribution and variance explained are different quantities. Uncovered intervals contributed zero deficit in the primary calculation.

### 2.5 Component and hemoglobin comparisons

At the first matched measurement, we calculated Pearson and Spearman correlations between CaO_2_ and hemoglobin, *R*^2^ from CaO_2_ regressed on hemoglobin alone, and the percentage of CaO_2_ contributed by dissolved oxygen: 100 × (0.003 × PaO_2_)/CaO_2_. We also measured how often hemoglobin was <9 or <10 g/dL among patients with low CaO_2_, including those whose SpO_2_ remained ≥92%. For a fair longitudinal comparison, the MIMIC-IV lower quartile of first hemoglobin (9.10 g/dL) was fixed before use in eICU. We then calculated a matched hemoglobin burden at the same blood-gas time points, with the same duplicate handling, 6-h limit, 24-h denominator, and uncovered-time rule as the CaO_2_ burden. Hemoglobin <10 g/dL was examined as a second, clinically familiar threshold.

### 2.6 Outcomes, phenotype, and covariates

Hospital mortality, defined as death before hospital discharge, was the primary outcome. ICU mortality was death before discharge from the index ICU. Furthermore, 28-day in-hospital mortality was a recorded hospital death within 28 days of ICU admission; deaths after hospital discharge were not available. Preserved-SpO_2_ low content was defined at the first matched measurement as SpO_2_ ≥ 92% and CaO_2_ < 12.48 mL O_2_/dL. This phenotype was descriptive and secondary (Figure 3). The main case-mix model included age; sex; harmonized race/ethnicity; and diagnosis-derived indicators for sepsis, pneumonia, chronic obstructive pulmonary disease, heart failure, chronic kidney disease, liver disease, malignancy, diabetes, and bleeding. These hospitalization-level indicators describe the case mix but are not verified pre-ICU comorbidities.

**Figure 3:**
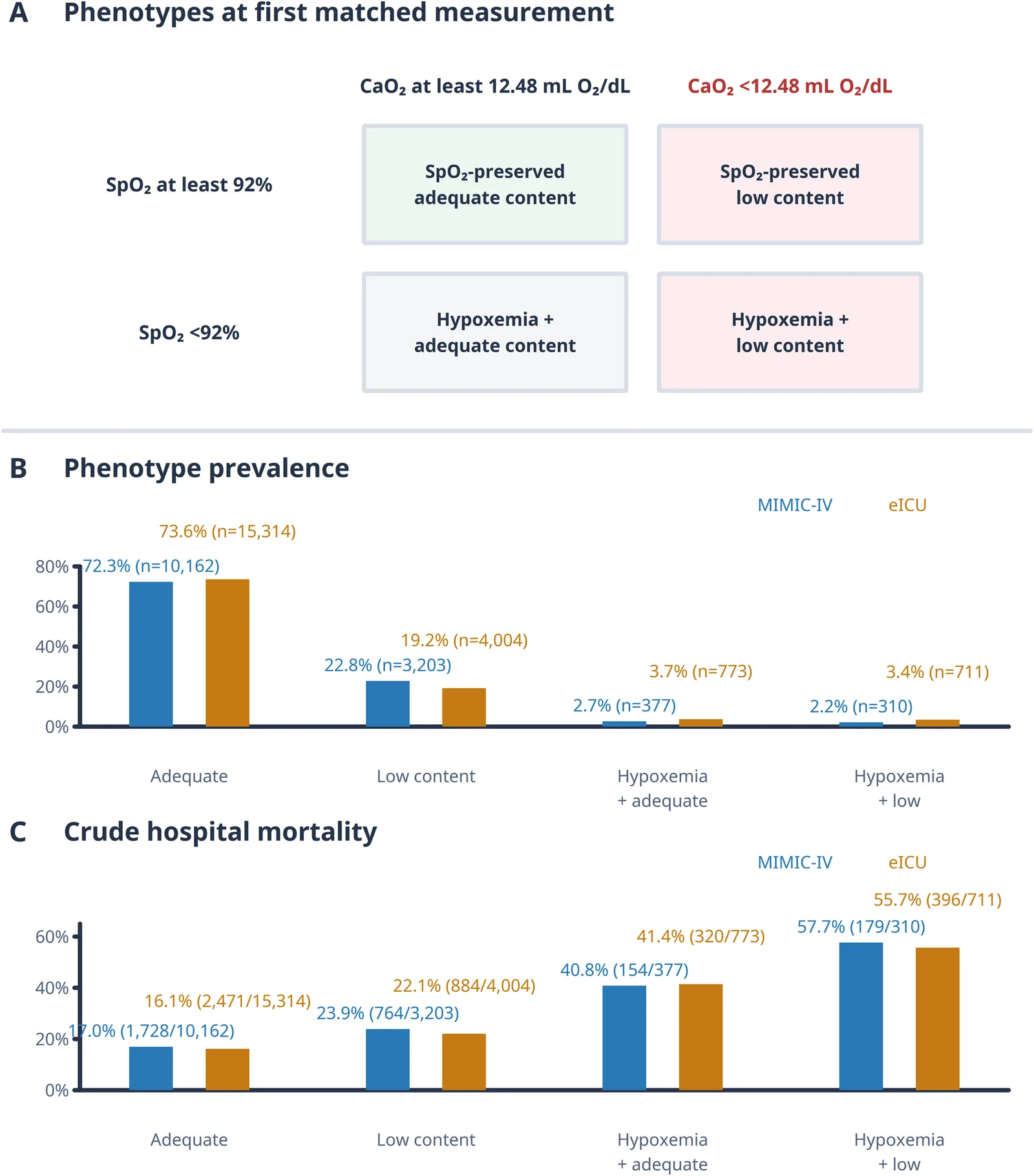
Phenotype prevalence and crude hospital mortality at the first matched measurement. **(A)** Classification of the four phenotypes according to SpO2 and CaO2 at the first matched measurement. **(B)** Phenotype prevalence in MIMIC-IV and eICU. **(C)** Crude hospital mortality according to phenotype in MIMIC-IV and eICU. Phenotype comparisons are descriptive.

### 2.7 Prespecified adjustment hierarchy

Our adjustment strategy was guided by the timing of covariates. Age and sex clearly preceded the exposure, while diagnosis indicators were retained as broad case-mix descriptors. Red-cell transfusion, vasopressor use, renal replacement therapy, and maximum fraction of inspired oxygen (FiO_2_) during the first 24 h could occur in response to anemia, hypoxemia, or worsening illness while the burden was accumulating. Adjusting for them may control some severity differences but could also remove part of the pathway or introduce collider bias. We therefore treated them as sensitivity covariates, not as the main adjustment set. In eICU, we added the Acute Physiology and Chronic Health Evaluation (APACHE) score in a key severity-adjusted analysis and used hospital-cluster robust inference. We retained the symmetric demographic and diagnosis-derived case-mix model as the primary comparison because a directly comparable validated MIMIC-IV severity score was not available in the prespecified derived tables; the APACHE estimate is nevertheless reported prominently because it addresses major residual severity confounding.

### 2.8 Matched hemoglobin and parsimony models

All head-to-head models used identical patients and covariates. Model H included the matched hemoglobin burden, Model C included the CaO_2_ burden, and Model HC included both. We repeated these comparisons for the 9.10 and 10 g/dL hemoglobin thresholds. We calculated variance inflation factors (VIFs) for both exposure terms in each HC design matrix. Because the burdens were highly correlated, the HC coefficients were used to demonstrate instability from collinearity and were not interpreted as independent physiologic effects. For a separate parsimony comparison, Model D combined first and 24-h summary measures of hemoglobin, SpO_2_, and PaO_2_ with one CaO_2_ burden variable. Model E replaced that single burden with three separate deficit burdens for hemoglobin <10 g/dL, SpO_2_ < 92%, and PaO_2_ < 60 mmHg.

### 2.9 Landmark analysis, exposure scale, and statistical methods

The 24-h landmark analysis included only patients who were alive and still in the index ICU at hour 24. Exposure was fixed using measurements from hours 0–24, and only deaths after the landmark were counted. Logistic regression produced model-based confidence intervals in MIMIC-IV and finite-sample-corrected hospital-cluster robust intervals in eICU. Continuous burden was reported per database-specific SD and per fixed 0.5 mL O_2_/dL increase. Because many patients had zero burden, we also compared zero burden with three groups formed from the positive-burden distribution. Cutoff points were calculated once in each primary cohort and then reused in sensitivity and landmark samples. We did not assume a monotonic trend.

Prediction metrics were exploratory and are reported in the Supplementary Material. Akaike information criterion and Bayesian information criterion values came from full-sample fits. Area under the receiver operating characteristic curve (AUROC) and Brier scores were estimated with deterministic stratified fivefold cross-validation. We did not perform frozen-coefficient external prediction validation because the study was not designed to develop a prediction model. We also did not perform decision-curve analysis, machine learning models, restricted cubic splines, or broad data-driven subgroup searches. Tests were two-sided, and interpretation focused on effect size, consistency, and sensitivity to modeling choices rather than on statistical significance alone.

After editorial review, we performed two explicitly *post hoc* analyses. We stratified patients by the first matched SpO_2_ (<90%, 90%–94%, or ≥95%) and repeated the first-anchor component correlations and matched H-versus-C fivefold cross-validated AUROC and Brier comparisons within each stratum. Using the same out-of-fold predictions in the full paired samples, we also calculated observed and mean predicted mortality, the observed-to-expected ratio, calibration-in-the-large, the joint calibration intercept and slope, and observed mortality by predicted-risk decile. These analyses did not alter the cutoff, primary model, or prespecified conclusions.

### 2.10 Missing data, measurement coverage, and repeated patients

Eligibility required a matched PaO_2_, hemoglobin, and SpO_2_ measurement, so the first-measurement cohort was complete for those variables by design. The main burden model used complete cases for burden and model covariates. Age and diagnosis indicators were complete in the analysis cohort, and unknown sex or race/ethnicity values were retained as categories; only 2 MIMIC-IV and 22 eICU stays lacked repeated-measure burden. Analyses that added FiO_2_, treatment variables, direct SaO_2_, or APACHE used the available complete cases, with denominators reported in Supplementary Tables S6 and S7. We did not impute intermittently measured physiologic variables because missingness reflected clinical measurement decisions and was unlikely to be random (25). To examine this observation process, we adjusted for coverage hours, time of the first eligible value, and record count. We also calculated the mean deficit only over observed time among stays with at least 6 or 12 h of coverage. The same interval rules were applied in eICU: values were not interpolated beyond 6 h, and uncovered time contributed zero deficit rather than an assumed low value. In eICU, a one-observation-per-patient analysis retained one hospitalization per unique patient using the earliest available hospitalization year and then the lowest unit-stay identifier.

### 2.11 Software and reproducibility

Analyses were performed in Python 3.13 using pandas, NumPy, SciPy, statsmodels, and scikit-learn. Figures were generated in R 4.5.2 using grid, svglite, and ragg. VIFs were calculated from the same design matrices used in the joint Hb–CaO_2_ models. Model denominators, hospital counts, exposure cut points, and aggregate figure data were exported before manuscript assembly. The submission code contains cohort-extraction templates, exposure and model scripts, aggregate source data, and an aggregate-only figure renderer. Patient-level derived data were not redistributed under the database-use agreements.

## 3 Results

### 3.1 Cohorts and component analysis

Of the upstream invasive-ventilation cohorts, 14,052 of 23,811 MIMIC-IV stays and 20,802 of 38,416 eICU stays had the matched measurements required to calculate the first CaO_2_ value and a known hospital outcome. This corresponds to 41.0% and 45.9% not entering the first-measurement cohorts, respectively. Repeated-measure burden was available for 14,050 and 20,780 stays, respectively; hospital mortality was 20.1% (2,825/14,052) and 19.6% (4,071/20,802), respectively (Figure 1; Table 1). Median age was 64 and 65 years, and women comprised 40.1% and 41.5%, respectively. Median first SpO_2_ was 100% and 99%, median hemoglobin was 10.6 and 10.9 g/dL, and median CaO_2_ was 14.49 and 14.74 mL O_2_/dL, respectively. Median measurement coverage was 12.0 h in MIMIC-IV and 10.08 h in eICU.

**Table 1:** Baseline characteristics of the primary phenotype cohorts.

Characteristic	MIMIC-IV primary cohort	eICU primary cohort
Patients, *n*	14,052	20,802
Hospital mortality, *n* (%)	2,825 (20.1)	4,071 (19.6)
ICU mortality, *n* (%)	2,284 (16.3)	2,987 (14.4)
28-day in-hospital mortality, *n* (%)	2,695 (19.2)	3,971 (19.1)
Age, years, median [IQR]	64.0 [53.0, 75.0]	65.0 [54.0, 75.0]
Female sex, *n* (%)	5,637 (40.1)	8,634 (41.5)
Race/ethnicity: White, *n* (%)	8,585 (61.1)	15,901 (76.4)
Race/ethnicity: Black, *n* (%)	1,033 (7.4)	2,222 (10.7)
Race/ethnicity: Asian, *n* (%)	373 (2.7)	400 (1.9)
Race/ethnicity: Hispanic, *n* (%)	455 (3.2)	813 (3.9)
Race/ethnicity: Other/unknown, *n* (%)	3,606 (25.7)	1,466 (7.0)
First hemoglobin, g/dL, median [IQR]	10.60 [9.10, 12.20]	10.90 [9.40, 12.50]
First PaO_2_, mmHg, median [IQR]	140.00 [77.00, 252.00]	121.00 [84.00, 191.00]
First SpO₂, %, median [IQR]	100.00 [97.00, 100.00]	99.00 [97.00, 100.00]
First SpO_2_-derived CaO₂, mL O_2_/dL, median [IQR]	14.49 [12.49, 16.60]	14.74 [12.69, 16.88]
First-24-h CaO₂ deficit burden, median [IQR]	0.00 [0.00, 0.15]	0.00 [0.00, 0.13]
Exposure records, *n*, median [IQR]	3.00 [2.00, 5.00]	2.00 [1.00, 3.00]
Exposure coverage, h, median [IQR]	12.00 [7.93, 17.58]	10.08 [6.00, 13.62]
Lactate maximum, mmol/L, median [IQR]	2.20 [1.50, 3.50]	2.40 [1.40, 4.79]
FiO_2_ maximum, median [IQR]	0.90 [0.50, 1.00]	0.60 [0.50, 1.00]
SpO₂-preserved low oxygen content, *n* (%)	3,203 (22.8)	4,004 (19.2)
Hypoxemia plus low oxygen content, *n* (%)	310 (2.2)	711 (3.4)
Vasopressor use in first 24 h, *n* (%)	8,373 (59.6)	9,464 (45.5)
RBC transfusion in first 24 h, *n* (%)	3,403 (24.2)	1,365 (6.6)
Renal replacement therapy in first 24 h, *n* (%)	438 (3.1)	809 (3.9)
MIMIC physiologic severity proxy, median [IQR]	0.44 [−0.78, 1.54]	—
eICU APACHE score, median [IQR]	—	72.00 [54.00, 96.00]
eICU acute physiology score, median [IQR]	—	60.00 [42.00, 84.00]

Values are *n* (%) or median [IQR], unless otherwise stated. CaO_2_ burden is the time-weighted mean deficit below 12.48 mL O_2_/dL over ICU hours 0–24.

CaO_2_ was strongly dominated by hemoglobin in both databases. The Pearson correlation values between the first CaO_2_ value and hemoglobin were 0.965 in MIMIC-IV and 0.949 in eICU; the Spearman correlation values were 0.967 and 0.958, respectively. Hemoglobin alone explained *R*^2^ values of 0.932 and 0.901, respectively. Dissolved oxygen contributed a median of 2.9% (IQR 1.6%–5.3%) and 2.5% (1.7%–4.0%) of CaO_2_, respectively (Figure 2D; Supplementary Table S8). Among patients below the CaO_2_ cutoff, 84.7% and 79.5% had hemoglobin <9 g/dL, and 97.8% and 95.4% had hemoglobin <10 g/dL, respectively. Among patients with preserved SpO_2_ but low CaO_2_, 88.4% and 85.8% had hemoglobin <9 g/dL, respectively, and almost all had hemoglobin <10 g/dL. The CaO_2_ and matched 9.10 g/dL hemoglobin burdens were also highly correlated (Pearson *r* = 0.949 and 0.919, respectively).

### 3.2 Adjustment hierarchy

In the demographic and diagnosis-derived case-mix model, the odds ratio for hospital mortality per 1-SD higher 24-h low-CaO_2_ burden was 1.33 (95% confidence interval 1.28–1.39) in MIMIC-IV and 1.29 (1.24–1.35) in eICU, with robust inference across 180 hospitals (Figure 4; Table 2). Adding APACHE score in eICU attenuated the estimate to 1.14 (1.10–1.19) across 167 hospitals, showing that illness severity explained a substantial part of the association. Estimates from the concurrent treatment and FiO_2_ sensitivity models were 1.36 (1.30–1.42) in MIMIC-IV and 1.23 (1.17–1.28) in eICU across 147 hospitals. Because these covariates could be downstream of the exposure or evolving illness, we treated these models as sensitivity analyses.

**Figure 4:**
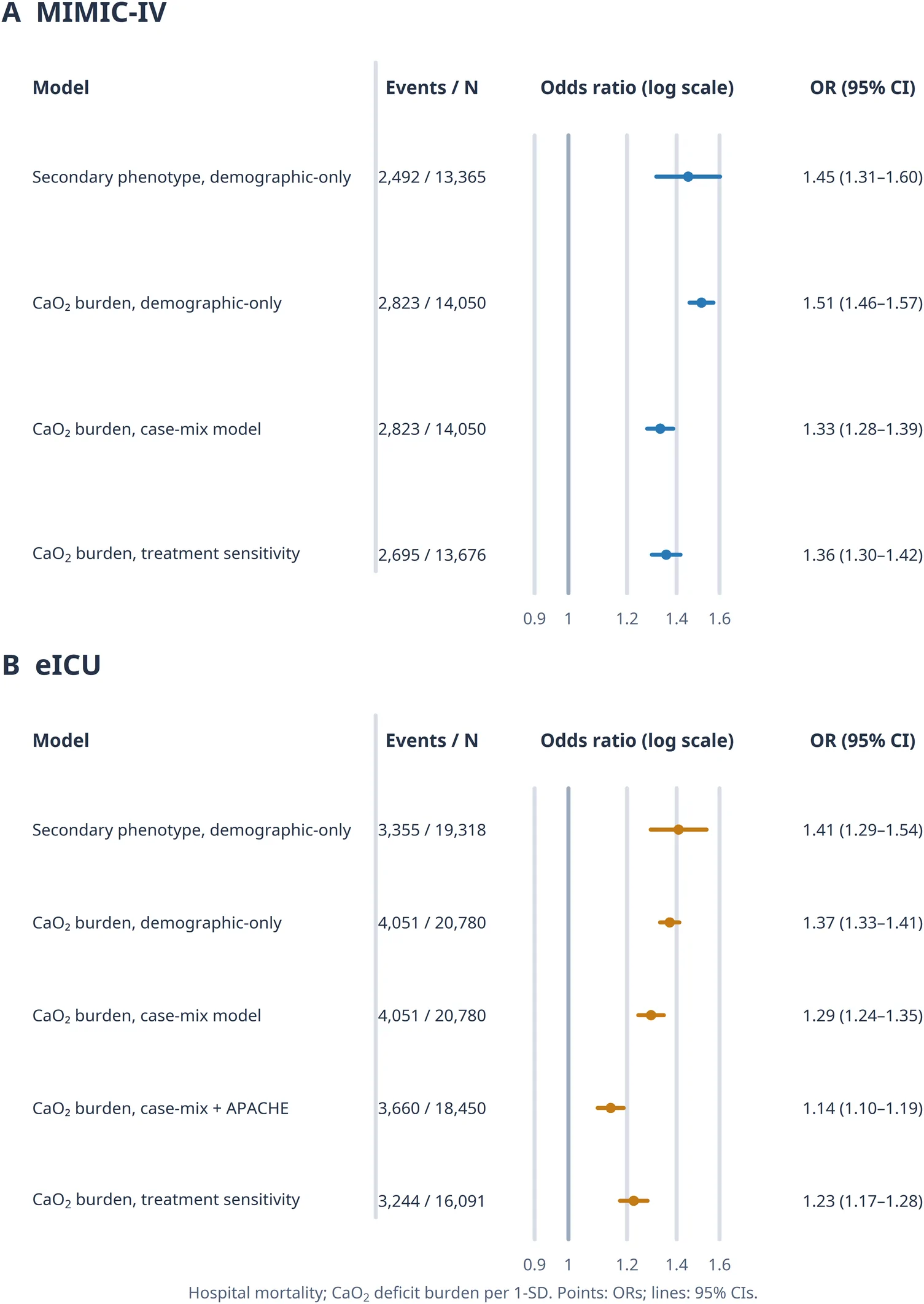
Adjusted associations with hospital mortality. **(A)** MIMIC-IV. **(B)** eICU. Burden odds ratios are per database-specific 1-SD increase. eICU estimates use hospital-cluster robust inference where indicated.

**Table 2:** Adjustment hierarchy for CaO_2_ deficit burden.

Database	Adjustment set	*N*	Events	OR (95% CI)	SE approach	Hospital clusters
MIMIC-IV	Demographic and diagnosis-derived case-mix	14,050	2,823	1.33 (1.28–1.39)	Model-based	
MIMIC-IV	Concurrent treatment/FiO_2_ sensitivity	13,676	2,695	1.36 (1.30–1.42)	Model-based	
eICU	Demographic and diagnosis-derived case-mix	20,780	4,051	1.29 (1.24–1.35)	Hospital-cluster robust	180
eICU	Base + APACHE	18,450	3,660	1.14 (1.10–1.19)	Hospital-cluster robust	167
eICU	Concurrent treatment/FiO_2_ sensitivity	16,091	3,244	1.23 (1.17–1.28)	Hospital-cluster robust	147

Odds ratios are per database-specific 1-SD increase in 24-h low-CaO_2_ burden. Diagnosis indicators are hospitalization-level case-mix descriptors. Concurrent-treatment models additionally include first-24-h RBC transfusion, vasopressor use, renal replacement therapy, and maximum FiO_2_ and are sensitivity analyses. RBC, red blood cell; SE, standard error.

### 3.3 Comparison with matched hemoglobin burden

The MIMIC-IV lower quartile for first hemoglobin was 9.10 g/dL and was applied unchanged to eICU. In identical case-mix samples, the matched hemoglobin-burden model had odds ratios of 1.21 in MIMIC-IV and 1.15 in eICU, compared with 1.33 and 1.29 for the CaO_2_-burden model, respectively. Cross-validated AUROC was 0.742 versus 0.748 in MIMIC-IV and 0.667 versus 0.677 in eICU, differences of only 0.006 and 0.010, respectively. In the model containing both burdens, exposure-specific VIFs were 10.25 for hemoglobin burden and 10.40 for CaO_2_ burden in MIMIC-IV, and 6.51 and 6.53 in eICU. The two coefficients moved in opposite directions, confirming that the joint estimates were unstable because of collinearity; they were not evidence of two independent effects. Results using hemoglobin <10 g/dL led to the same interpretation (Supplementary Tables S9, S11, and S15).

### 3.4 Landmark and exposure scale

The 24-h landmark retained 12,956 MIMIC-IV and 18,325 eICU stays, excluding 1,094 and 2,455 patients who died or left the index ICU before hour 24, respectively. Adjusted odds ratios per 1-SD were 1.26 (1.21–1.32) and 1.19 (1.14–1.25), respectively (Supplementary Table S10). The burden SD was 0.456 mL O_2_/dL in MIMIC-IV and 0.400 mL O_2_/dL in eICU. On a common scale, odds ratios per 0.5 mL O_2_/dL were 1.37 (1.31–1.43) and 1.38 (1.31–1.45), respectively. In categorical analyses, the highest positive-burden group had the clearest increase in risk, while lower positive groups were not consistently above the zero-burden reference. The data therefore did not support a simple monotonic dose-response across all non-zero values.

### 3.5 Measurement coverage, repeated patients, and alternative cutoffs

After adjustment for coverage hours, time of the first eligible value, and record count, odds ratios per 1-SD burden were 1.31 (1.25–1.36) in MIMIC-IV and 1.29 (1.24–1.33) in eICU. Analyses restricted to at least 6 h of coverage gave 1.35 (1.29–1.40) and 1.33 (1.28–1.37), respectively; with at least 12 h, estimates were 1.29 (1.22–1.37) and 1.24 (1.17–1.31), respectively (Supplementary Table S12). These checks reduce, but do not remove, concern about selective measurement and uncovered time. The eICU cohort contained 20,802 stays from 20,090 patients. Restricting to one stay per patient produced case-mix, APACHE, and landmark estimates of 1.30 (1.25–1.36), 1.14 (1.10–1.19), and 1.20 (1.15–1.26), respectively (Supplementary Table S13). Alternative P20, P25, and P33 thresholds and numerical cutoffs of 14, 15, and 16 mL O_2_/dL, respectively, all produced positive estimates in both databases, although the association weakened as the eICU numerical cutoff increased and was 1.05 (1.00–1.09) at 16 mL O_2_/dL (Supplementary Table S3). This sensitivity supports a lower-tail association but not the clinical superiority of the 12.48 cutoff.

### 3.6 Parsimony and exploratory model performance

Model D used one CaO_2_ burden variable in addition to first and 24-h component summaries. Model E instead used three component-specific burdens. Their cross-validated performance was nearly identical: D-minus-E AUROC differences were −0.0005 in MIMIC-IV and −0.0012 in eICU, and Brier differences were 0.00002 and 0.00031, respectively. Thus, the combined metric did not improve prediction over detailed component histories. Its narrower advantage is that one variable summarizes threshold depth and duration in familiar physiologic units. Direct-SaO_2_, longer-window, record-count, coverage, and other prespecified sensitivity analyses were directionally consistent (Figure 5; Supplementary Tables S2–S5).

**Figure 5:**
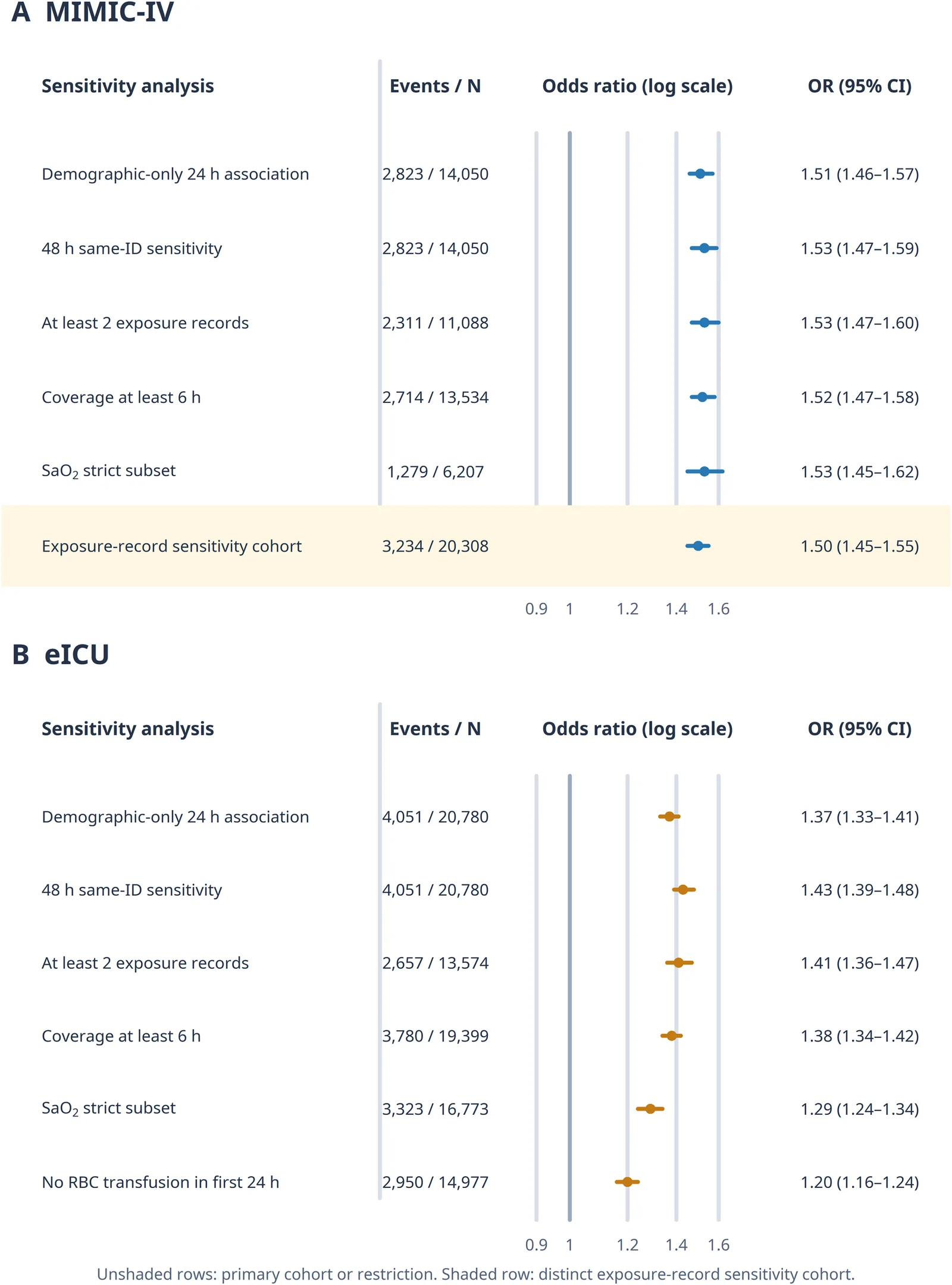
Prespecified sensitivity analyses. **(A)** MIMIC-IV. **(B)** eICU. The shaded row uses a distinct exposure-record sensitivity cohort.

### 3.7 *Post hoc* SpO_2_-stratified and calibration analyses

At first SpO_2_ < 90%, CaO_2_–hemoglobin Pearson correlation values were 0.887 in MIMIC-IV and 0.826 in eICU, and hemoglobin explained *R*^2^ values of 0.787 and 0.682, respectively. The paired model samples contained 399/208 and 1,022/541 stays/deaths, respectively; CaO_2_-minus-hemoglobin fivefold cross-validated AUROC differences were 0.045 and 0.044, respectively. At first SpO_2_ ≥ 95%, correlations were 0.987 and 0.989, *R*^2^ values were 0.973 and 0.978, and corresponding AUROC differences were only 0.002 and 0.002, respectively. Results for the intermediate 90%–94% stratum were also close to the preserved-saturation estimates (Supplementary Table S16). These *post hoc* findings show that hemoglobin dominance was less complete during marked desaturation, but they do not establish subgroup-specific clinical superiority.

In the full paired samples, out-of-fold calibration slopes for the hemoglobin and CaO_2_ models were 0.983 and 0.983 in MIMIC-IV and 0.982 and 0.984 in eICU. Observed-to-expected ratios ranged from 0.999 to 1.000, and calibration-in-the-large was within 0.001 of zero for all four model-database combinations (Supplementary Table S17). Calibration was therefore similar for the two burden models and did not indicate a clinically meaningful predictive advantage for CaO_2_.

## 4 Discussion

### 4.1 Principal findings

A greater 24-h low-CaO_2_ burden was associated with hospital mortality in MIMIC-IV, and the association was reproduced in eICU. The estimate weakened after APACHE adjustment and after the 24-h landmark but remained positive. The component and head-to-head analyses are central to interpretation: CaO_2_ was overwhelmingly driven by hemoglobin, the majority of preserved-SpO_2_ low-content patients were anemic, the CaO_2_ model improved AUROC only slightly over a matched hemoglobin model, and it did not outperform detailed component histories. These findings support a compact prognostic summary, not a new independent physiologic signal. The *post hoc* saturation-stratified analysis refined this interpretation: hemoglobin dominance was nearly complete when first SpO_2_ was ≥95% but was less complete when first SpO_2_ was <90%.

### 4.2 Relation to prior evidence

The basic physiology in this study is established: hemoglobin carries most arterial oxygen, and adequate saturation does not guarantee adequate oxygen content. Harvey and colleagues previously related time-weighted CaO_2_ exposure to mortality, while other investigators studied SpO_2_/FiO_2_ time at risk, PaO_2_ exposure, time-varying oxygen intensity, and hyperoxia (4–12). Our more modest contribution is methodological and comparative. We focused on deficit below a prespecified lower-tail cutoff, combined deficit depth with duration, applied the MIMIC-IV cutoff unchanged in eICU, and directly compared the result with a hemoglobin burden built on the same time grid. This is an observational summary; oxygen-target and transfusion trials have addressed treatment strategies (26–38). Our findings therefore cannot define an oxygen or transfusion threshold.

### 4.3 Physiologic interpretation and clinical relevance

The component results closely followed the CaO_2_ equation. SpO_2_ was usually near the top of its measurement range, while dissolved oxygen accounted for only a few percent of content. The majority of the between-patient variation therefore came from hemoglobin. This interpretation applies most directly to the large majority with preserved saturation; in the *post hoc* first-SpO_2_ < 90% strata, the CaO_2_–hemoglobin correlation was lower, consistent with a larger contribution from saturation variation. Preserved SpO_2_ with low CaO_2_ is best understood here as reduced oxygen-carrying capacity despite apparently satisfactory saturation, usually because of anemia. Pulse-oximetry error remains important during desaturation and for phenotype classification, but it is unlikely to explain most of a measure whose variation was dominated by hemoglobin (13–19).

The CaO_2_ burden still offers a concise description: it expresses how far content fell below a cutoff and for how long, in one physiologic unit. However, the AUROC advantage over one matched hemoglobin threshold was small, and there was no advantage over three detailed component burdens. We therefore do not regard it as an independent biomarker or a better prediction model. CaO_2_ is also not systemic oxygen delivery. We did not measure cardiac output, regional perfusion, microcirculatory flow, diffusion, tissue extraction, or oxygen consumption comprehensively. A high burden should not be equated with tissue oxygen debt.

### 4.4 Covariate timing, landmark correction, and limitations

The adjustment hierarchy also limits interpretation. Hospitalization-level diagnosis indicators may not precede the exposure and cannot remove residual confounding. Transfusion, vasopressors, renal replacement therapy, and FiO_2_ during the same 24-h window may reflect both evolving illness and responses to anemia or hypoxemia. Their inclusion can reduce some severity imbalance but can also over-adjust or condition on common consequences. The attenuation after APACHE adjustment in eICU suggests that illness severity explains a substantial part of the association and is central to the clinical interpretation. The absence of a comparable validated MIMIC-IV score limits symmetry between databases.

The landmark analysis reduced concern that early death alone created the association, but it selected patients who survived and remained in the ICU for 24 h and therefore answers a different question (39). Measurement and selection are additional concerns. Requiring temporally matched PaO_2_, hemoglobin, and SpO_2_ excluded 41.0% of upstream MIMIC-IV stays and 45.9% of upstream eICU stays. These patients may have been monitored differently, and the locked aggregate flow did not preserve mutually exclusive exclusion reasons. Among included patients, sicker patients may have had more frequent blood gases. Coverage adjustment and observed-time analyses were reassuring but cannot eliminate surveillance bias.

Other limitations include unmeasured confounding; possible misclassification from diagnosis records and database-specific ventilation definitions; pulse-oximetry rounding, ceiling effects, and differential error; incomplete direct SaO_2_ data; and repeated eICU hospitalizations in the main stay-level analysis. The 6-h representation limit is pragmatic rather than biological. Assigning zero deficit to uncovered time avoids imputing an unobserved low value but can underestimate burden, especially if low values lead to more frequent testing. The 12.48 mL O_2_/dL cutoff is an empirical MIMIC-IV lower quartile, not an established clinical boundary, and its effect size varied across alternative cutoffs. Applying it unchanged to eICU assumes that one numerical lower-tail definition remains informative despite differences in case mix, blood-gas sampling, and practice patterns. Median first SpO_2_ was 100% in MIMIC-IV and 99% in eICU, and only 401 and 1,040 first-anchor observations were <90%, respectively; therefore, the overall hemoglobin-dominance conclusion may not generalize to sustained severe hypoxemia or selected acute respiratory distress syndrome populations. The more favorable *post hoc* CaO_2_-versus-hemoglobin comparison in the <90% strata is hypothesis-generating and does not establish subgroup-specific clinical superiority. Postdischarge deaths were unavailable. Finally, observational associations cannot show that changing oxygen, ventilation, transfusion, or measurement frequency would improve outcomes.

### 4.5 Implications

For clinicians, the main message is limited but clear: preserved SpO_2_ does not necessarily mean normal arterial oxygen content, and in these cohorts, low content usually reflected low hemoglobin. The burden may be useful in research as one number describing the depth and duration of low content. It should not trigger a specific oxygen or transfusion intervention. Future studies should use standardized measurement schedules, test the cutoff in other health systems, and add cardiac output or perfusion measurements when feasible before considering any clinical application.

## 5 Conclusion

Low CaO_2_ burden during the first ICU day was associated with hospital mortality in MIMIC-IV and eICU, but the eICU estimate was substantially attenuated after APACHE adjustment. In these cohorts, which were dominated by preserved oxygen saturation, the measure was predominantly driven by hemoglobin and offered only small predictive differences compared with a matched hemoglobin burden, with no advantage over detailed component histories. *Post hoc* findings suggest that hemoglobin dominance may be less complete during marked desaturation and require confirmation in severely hypoxemic populations. The metric's most defensible role is as a compact observational summary of how far and for how long arterial oxygen content was below an empirical cutoff. It is not an independent measure of tissue oxygenation and does not support changing oxygen or transfusion targets.

## 6 Code availability statement

Cohort extraction templates, Python exposure and model-fitting scripts, aggregate source data, sensitivity analysis code, and the aggregate-only R figure renderer are provided as Supplementary Code. The code package contains no patient-level data or local filesystem paths.

## Data Availability

The datasets analyzed in this study are MIMIC-IV version 3.1 and eICU version 2.0, which are available from PhysioNet to credentialed investigators after completion of the required human-subjects research training and applicable data use agreements. Patient-level derived data cannot be redistributed under the database use agreements. Analysis code and aggregate source data are provided in the Supplementary Material. Further inquiries can be directed to the corresponding authors.
