# Supplementary material for: Low arterial oxygen-content burden during the first 24 h and hospital mortality in mechanically ventilated adults: analyses of MIMIC-IV and eICU
Source: Front Med (Lausanne). 2026 Oct 9;13:1935082. doi: 10.3389/fmed.2026.1935082 (PMC13690817; doi:10.3389/fmed.2026.1935082)
Supplement: Supplementary file 1 [file Table1.docx]

Supplementary Tables S1–S17

**Table S1. Baseline characteristics by preserved-SpO₂ phenotype**

| **Database** | **Characteristic** | **Adequate content** | **Low content** |
| --- | --- | --- | --- |
| MIMIC-IV | Patients, n | 10,162 | 3,203 |
| MIMIC-IV | Hospital mortality, n (%) | 1,728 (17.0) | 764 (23.9) |
| MIMIC-IV | Age, years | 63.0 [52.0, 74.0] | 66.0 [55.0, 77.0] |
| MIMIC-IV | First hemoglobin, g/dL | 11.30 [10.20, 12.70] | 8.10 [7.40, 8.60] |
| MIMIC-IV | First PaO₂, mmHg | 150.00 [84.00, 257.00] | 134.00 [69.00, 260.00] |
| MIMIC-IV | First SpO₂, % | 100.00 [97.00, 100.00] | 100.00 [97.00, 100.00] |
| MIMIC-IV | First SpO₂-derived CaO₂, mL O₂/dL | 15.48 [14.03, 17.29] | 11.23 [10.33, 11.90] |
| MIMIC-IV | First-24h CaO₂ deficit burden | 0.00 [0.00, 0.00] | 0.43 [0.15, 0.93] |
| MIMIC-IV | Lactate maximum, mmol/L | 2.20 [1.50, 3.30] | 2.40 [1.50, 3.80] |
| MIMIC-IV | FiO₂ maximum | 0.70 [0.50, 1.00] | 1.00 [0.50, 1.00] |
| MIMIC-IV | Vasopressor use, n (%) | 5,726 (56.3) | 2,116 (66.1) |
| MIMIC-IV | RBC transfusion, n (%) | 1,698 (16.7) | 1,562 (48.8) |
| eICU | Patients, n | 15,314 | 4,004 |
| eICU | Hospital mortality, n (%) | 2,471 (16.1) | 884 (22.1) |
| eICU | Age, years | 64.0 [53.0, 74.0] | 67.0 [56.0, 76.0] |
| eICU | First hemoglobin, g/dL | 11.50 [10.40, 12.90] | 8.20 [7.50, 8.70] |
| eICU | First PaO₂, mmHg | 128.00 [89.00, 199.00] | 114.00 [81.00, 174.00] |
| eICU | First SpO₂, % | 100.00 [97.00, 100.00] | 100.00 [97.00, 100.00] |
| eICU | First SpO₂-derived CaO₂, mL O₂/dL | 15.67 [14.14, 17.52] | 11.30 [10.42, 11.95] |
| eICU | First-24h CaO₂ deficit burden | 0.00 [0.00, 0.00] | 0.46 [0.20, 0.83] |
| eICU | Lactate maximum, mmol/L | 2.30 [1.40, 4.10] | 2.30 [1.20, 5.50] |
| eICU | FiO₂ maximum | 0.60 [0.50, 1.00] | 0.60 [0.50, 1.00] |
| eICU | Vasopressor use, n (%) | 6,465 (42.2) | 2,028 (50.6) |
| eICU | RBC transfusion, n (%) | 615 (4.0) | 625 (15.6) |

*Note: Values are descriptive. Preserved SpO₂ was defined as SpO₂ ≥92%; low CaO₂ was defined as <12.48 mL O₂/dL.*

**Table S2. Prespecified sensitivity analyses**

| **Database** | **Cohort source** | **Adjustment set** | **Analysis** | **Exposure** | **N** | **Events** | **OR (95% CI)** | **P value** |
| --- | --- | --- | --- | --- | --- | --- | --- | --- |
| MIMIC-IV | Primary 24 h analytic cohort | Age, sex, race/ethnicity | Primary first-24h SpO₂-derived burden | CaO₂ deficit burden, per 1-SD increase | 14050 | 2823 | 1.51 (1.46-1.57) | <0.001 |
| eICU | Primary 24 h analytic cohort | Age, sex, race/ethnicity | Primary first-24h SpO₂-derived burden | CaO₂ deficit burden, per 1-SD increase | 20780 | 4051 | 1.37 (1.33-1.41) | <0.001 |
| MIMIC-IV | 48 h same-ID sensitivity from the primary 24 h cohort | Age, sex, race/ethnicity | 48h same-ID sensitivity | CaO₂ deficit burden, per 1-SD increase | 14050 | 2823 | 1.53 (1.47-1.59) | <0.001 |
| MIMIC-IV | Primary 24 h analytic cohort restricted to ≥2 exposure records | Age, sex, race/ethnicity | At least 2 exposure records | CaO₂ deficit burden, per 1-SD increase | 11088 | 2311 | 1.53 (1.47-1.60) | <0.001 |
| MIMIC-IV | Primary 24 h analytic cohort restricted to coverage ≥6 h | Age, sex, race/ethnicity | Coverage at least 6h | CaO₂ deficit burden, per 1-SD increase | 13534 | 2714 | 1.52 (1.47-1.58) | <0.001 |
| MIMIC-IV | Primary 24 h analytic cohort restricted to the SaO₂ strict subset | Age, sex, race/ethnicity | SaO₂ strict subset | SaO₂-derived CaO₂ deficit burden, per 1-SD increase | 6207 | 1279 | 1.53 (1.45-1.62) | <0.001 |
| eICU | 48 h same-ID sensitivity from the primary 24 h cohort | Age, sex, race/ethnicity | 48h same-ID sensitivity | CaO₂ deficit burden, per 1-SD increase | 20780 | 4051 | 1.43 (1.39-1.48) | <0.001 |
| eICU | Primary 24 h analytic cohort restricted to ≥2 exposure records | Age, sex, race/ethnicity | At least 2 exposure records | CaO₂ deficit burden, per 1-SD increase | 13574 | 2657 | 1.41 (1.36-1.47) | <0.001 |
| eICU | Primary 24 h analytic cohort restricted to coverage ≥6 h | Age, sex, race/ethnicity | Coverage at least 6h | CaO₂ deficit burden, per 1-SD increase | 19399 | 3780 | 1.38 (1.34-1.42) | <0.001 |
| eICU | Primary 24 h analytic cohort restricted to the SaO₂ strict subset | Age, sex, race/ethnicity | SaO₂ strict subset | SaO₂-derived CaO₂ deficit burden, per 1-SD increase | 16773 | 3323 | 1.29 (1.24-1.34) | <0.001 |
| MIMIC-IV | Exposure-record sensitivity cohort, 24 h (distinct denominator) | Age, sex, race/ethnicity | Exposure-record sensitivity cohort | CaO₂ deficit burden, per 1-SD increase | 20308 | 3234 | 1.50 (1.45-1.55) | <0.001 |
| eICU | Exposure-record sensitivity cohort, 24 h (distinct denominator) | Age, sex, race/ethnicity | Exposure-record sensitivity cohort | CaO₂ deficit burden, per 1-SD increase | 20780 | 4051 | 1.37 (1.33-1.41) | <0.001 |
| MIMIC-IV | Refined transfusion sensitivity cohort (distinct denominator) | Age, sex, race/ethnicity + diagnosis-derived case-mix, treatment, FiO₂ + RBC event count | Adjusted for RBC event count | CaO₂ deficit burden, per 1-SD increase | 19898 | 3099 | 1.26 (1.21-1.31) | <0.001 |
| MIMIC-IV | Refined transfusion sensitivity cohort restricted to no RBC transfusion (distinct denominator) | Age, sex, race/ethnicity + diagnosis-derived case-mix, treatment, and FiO₂ | No RBC transfusion in first 24 h (refined 0-24 h definition) | CaO₂ deficit burden, per 1-SD increase | 14545 | 2304 | 1.24 (1.19-1.29) | <0.001 |
| eICU | Refined transfusion sensitivity cohort (distinct denominator) | Age, sex, race/ethnicity + diagnosis-derived case-mix, treatment, FiO₂ + RBC event count | Adjusted for RBC event count | CaO₂ deficit burden, per 1-SD increase | 16091 | 3244 | 1.22 (1.18-1.26) | <0.001 |
| eICU | Refined transfusion sensitivity cohort restricted to no RBC transfusion (distinct denominator) | Age, sex, race/ethnicity + diagnosis-derived case-mix, treatment, and FiO₂ | No RBC transfusion in first 24 h (refined 0-24 h definition) | CaO₂ deficit burden, per 1-SD increase | 14977 | 2950 | 1.20 (1.16-1.24) | <0.001 |
| MIMIC-IV | Primary burden cohort restricted to alive and still in the index ICU at 24 h | Age, sex, race/ethnicity + diagnosis-derived case-mix indicators | True 24-h landmark | CaO₂ deficit burden, 0–24 h fixed exposure, per 1-SD increase | 12956 | 2316 | 1.26 (1.21–1.32) | <0.001 |
| eICU | Primary burden cohort restricted to alive and still in the index ICU at 24 h | Age, sex, race/ethnicity + diagnosis-derived case-mix indicators | True 24-h landmark | CaO₂ deficit burden, 0–24 h fixed exposure, per 1-SD increase | 18325 | 3148 | 1.19 (1.14–1.25) | <0.001 |

**Table S3. Alternative CaO₂ cutoffs**

| **Database** | **Cohort source** | **Adjustment set** | **Cutoff** | **N** | **Events** | **OR (95% CI)** | **P value** |
| --- | --- | --- | --- | --- | --- | --- | --- |
| MIMIC-IV | Alternative-cutoff sensitivity cohort (distinct from the primary 24 h analytic denominator) | Age, sex, race/ethnicity + diagnosis-derived case-mix, treatment, and FiO₂ | P20 | 19898 | 3099 | 1.33 (1.28-1.39) | <0.001 |
| MIMIC-IV | Alternative-cutoff sensitivity cohort (distinct from the primary 24 h analytic denominator) | Age, sex, race/ethnicity + diagnosis-derived case-mix, treatment, and FiO₂ | P25 | 19898 | 3099 | 1.34 (1.29-1.39) | <0.001 |
| MIMIC-IV | Alternative-cutoff sensitivity cohort (distinct from the primary 24 h analytic denominator) | Age, sex, race/ethnicity + diagnosis-derived case-mix, treatment, and FiO₂ | P33 | 19898 | 3099 | 1.33 (1.28-1.39) | <0.001 |
| MIMIC-IV | Alternative-cutoff sensitivity cohort (distinct from the primary 24 h analytic denominator) | Age, sex, race/ethnicity + diagnosis-derived case-mix, treatment, and FiO₂ | 14 | 19898 | 3099 | 1.31 (1.26-1.36) | <0.001 |
| MIMIC-IV | Alternative-cutoff sensitivity cohort (distinct from the primary 24 h analytic denominator) | Age, sex, race/ethnicity + diagnosis-derived case-mix, treatment, and FiO₂ | 15 | 19898 | 3099 | 1.26 (1.20-1.31) | <0.001 |
| MIMIC-IV | Alternative-cutoff sensitivity cohort (distinct from the primary 24 h analytic denominator) | Age, sex, race/ethnicity + diagnosis-derived case-mix, treatment, and FiO₂ | 16 | 19898 | 3099 | 1.20 (1.15-1.26) | <0.001 |
| eICU | Alternative-cutoff sensitivity cohort (distinct from the primary 24 h analytic denominator) | Age, sex, race/ethnicity + diagnosis-derived case-mix, treatment, and FiO₂ | P20 | 16091 | 3244 | 1.25 (1.21-1.30) | <0.001 |
| eICU | Alternative-cutoff sensitivity cohort (distinct from the primary 24 h analytic denominator) | Age, sex, race/ethnicity + diagnosis-derived case-mix, treatment, and FiO₂ | P25 | 16091 | 3244 | 1.23 (1.18-1.27) | <0.001 |
| eICU | Alternative-cutoff sensitivity cohort (distinct from the primary 24 h analytic denominator) | Age, sex, race/ethnicity + diagnosis-derived case-mix, treatment, and FiO₂ | P33 | 16091 | 3244 | 1.19 (1.14-1.23) | <0.001 |
| eICU | Alternative-cutoff sensitivity cohort (distinct from the primary 24 h analytic denominator) | Age, sex, race/ethnicity + diagnosis-derived case-mix, treatment, and FiO₂ | 14 | 16091 | 3244 | 1.14 (1.10-1.19) | <0.001 |
| eICU | Alternative-cutoff sensitivity cohort (distinct from the primary 24 h analytic denominator) | Age, sex, race/ethnicity + diagnosis-derived case-mix, treatment, and FiO₂ | 15 | 16091 | 3244 | 1.09 (1.05-1.13) | <0.001 |
| eICU | Alternative-cutoff sensitivity cohort (distinct from the primary 24 h analytic denominator) | Age, sex, race/ethnicity + diagnosis-derived case-mix, treatment, and FiO₂ | 16 | 16091 | 3244 | 1.05 (1.00-1.09) | 0.029 |

*Note: The 12.48 mL O₂/dL threshold was the prespecified MIMIC-IV P25. Numerical cutoffs are sensitivity definitions, not treatment thresholds. These rows use a distinct alternative-cutoff sensitivity cohort.*

**Table S4. Database-specific ARDS and acute respiratory failure coding**

| **Characteristic** | **MIMIC-IV primary cohort** | **eICU primary cohort** |
| --- | --- | --- |
| ARDS/acute respiratory failure subset, n (%) | 6,637 (47.2) | 847 (4.1) |

**Table S5. Model D versus Model E parsimony comparison**

| **Database** | **Adjustment set** | **Model** | **N** | **Events** | **AIC** | **BIC** | **5-fold CV AUROC** | **5-fold CV Brier** | **Condition number** | **SE approach** | **Hospital clusters** |
| --- | --- | --- | --- | --- | --- | --- | --- | --- | --- | --- | --- |
| MIMIC-IV | Original parsimony comparison | D: window summaries + one CaO₂ burden | 14050 | 2823 | 12215.875096161064 | 12344.231516632017 | 0.7478762500741075 | 0.1378637869926851 | 25.89212618003885 | model-based |  |
| MIMIC-IV | Original parsimony comparison | E: window summaries + three component-deficit burdens | 14050 | 2823 | 12201.350149600425 | 12344.8073254209 | 0.748358394527438 | 0.1378472047595345 | 27.279660361791382 | model-based |  |
| MIMIC-IV | Original parsimony comparison | D minus E | 14050 | 2823 | 14.524946560641183 | -0.5758087888825685 | -0.0004821444533305 | 1.6582233150624903e-05 |  | descriptive contrast |  |
| eICU | Original parsimony comparison | D: window summaries + one CaO₂ burden | 20780 | 4051 | 18776.13999825 | 18927.033177278405 | 0.6830259519596659 | 0.141333858451402 | 94.40251674393186 | model-based |  |
| eICU | Original parsimony comparison | E: window summaries + three component-deficit burdens | 20780 | 4051 | 18734.79642152957 | 18901.573093087285 | 0.6842167440157421 | 0.1410281327738145 | 99.50395889589588 | model-based |  |
| eICU | Original parsimony comparison | D minus E | 20780 | 4051 | 41.34357672042461 | 25.46008419111968 | -0.0011907920560761 | 0.0003057256775874 |  | descriptive contrast |  |

**Table S6. Missingness and covariate availability**

| **Database** | **Domain** | **Variable** | **Denominator, n** | **Available, n** | **Missing, %** |
| --- | --- | --- | --- | --- | --- |
| MIMIC-IV | First anchor | First matched measurement SaO₂-derived CaO₂ availability | 14052 | 2261 | 83.9 |
| MIMIC-IV | Repeated exposure | Repeated-record SpO₂-derived CaO₂ burden | 14052 | 14050 | 0.0 |
| MIMIC-IV | Repeated exposure | Repeated-record SaO₂-derived CaO₂ burden | 14052 | 6207 | 55.8 |
| MIMIC-IV | Model covariate | Age | 14052 | 14052 | 0.0 |
| MIMIC-IV | Model covariate | Sex (unknown retained as category) | 14052 | 14052 | 0.0 |
| MIMIC-IV | Model covariate | Race/ethnicity (unknown retained as category) | 14052 | 14052 | 0.0 |
| MIMIC-IV | Model covariate | Sepsis indicator | 14052 | 14052 | 0.0 |
| MIMIC-IV | Model covariate | Pneumonia indicator | 14052 | 14052 | 0.0 |
| MIMIC-IV | Model covariate | Chronic obstructive pulmonary disease indicator | 14052 | 14052 | 0.0 |
| MIMIC-IV | Model covariate | Heart failure indicator | 14052 | 14052 | 0.0 |
| MIMIC-IV | Model covariate | Chronic kidney disease indicator | 14052 | 14052 | 0.0 |
| MIMIC-IV | Model covariate | Liver disease indicator | 14052 | 14052 | 0.0 |
| MIMIC-IV | Model covariate | Malignancy indicator | 14052 | 14052 | 0.0 |
| MIMIC-IV | Model covariate | Diabetes indicator | 14052 | 14052 | 0.0 |
| MIMIC-IV | Model covariate | Bleeding indicator | 14052 | 14052 | 0.0 |
| MIMIC-IV | Model covariate | Vasopressor use, first 24 h | 14052 | 14052 | 0.0 |
| MIMIC-IV | Model covariate | RBC transfusion, first 24 h | 14052 | 14052 | 0.0 |
| MIMIC-IV | Model covariate | Renal replacement therapy, first 24 h | 14052 | 14052 | 0.0 |
| MIMIC-IV | Model covariate | Maximum lactate, first 24 h | 14052 | 12508 | 11.0 |
| MIMIC-IV | Model covariate | Maximum FiO₂, first 24 h | 14052 | 13677 | 2.7 |
| MIMIC-IV | Model covariate | MIMIC-IV physiologic severity proxy | 14052 | 14052 | 0.0 |
| eICU | First anchor | First matched measurement SaO₂-derived CaO₂ availability | 20802 | 16454 | 20.9 |
| eICU | Repeated exposure | Repeated-record SpO₂-derived CaO₂ burden | 20802 | 20780 | 0.1 |
| eICU | Repeated exposure | Repeated-record SaO₂-derived CaO₂ burden | 20802 | 16773 | 19.4 |
| eICU | Model covariate | Age | 20802 | 20802 | 0.0 |
| eICU | Model covariate | Sex (unknown retained as category) | 20802 | 20802 | 0.0 |
| eICU | Model covariate | Race/ethnicity (unknown retained as category) | 20802 | 20802 | 0.0 |
| eICU | Model covariate | Sepsis indicator | 20802 | 20802 | 0.0 |
| eICU | Model covariate | Pneumonia indicator | 20802 | 20802 | 0.0 |
| eICU | Model covariate | Chronic obstructive pulmonary disease indicator | 20802 | 20802 | 0.0 |
| eICU | Model covariate | Heart failure indicator | 20802 | 20802 | 0.0 |
| eICU | Model covariate | Chronic kidney disease indicator | 20802 | 20802 | 0.0 |
| eICU | Model covariate | Liver disease indicator | 20802 | 20802 | 0.0 |
| eICU | Model covariate | Malignancy indicator | 20802 | 20802 | 0.0 |
| eICU | Model covariate | Diabetes indicator | 20802 | 20802 | 0.0 |
| eICU | Model covariate | Bleeding indicator | 20802 | 20802 | 0.0 |
| eICU | Model covariate | Vasopressor use, first 24 h | 20802 | 20802 | 0.0 |
| eICU | Model covariate | RBC transfusion, first 24 h | 20802 | 20802 | 0.0 |
| eICU | Model covariate | Renal replacement therapy, first 24 h | 20802 | 20802 | 0.0 |
| eICU | Model covariate | Maximum lactate, first 24 h | 20802 | 9118 | 56.2 |
| eICU | Model covariate | Maximum FiO₂, first 24 h | 20802 | 16108 | 22.6 |
| eICU | Model covariate | eICU APACHE score | 20802 | 18464 | 11.2 |
| eICU | Model covariate | eICU acute physiology score | 20802 | 18464 | 11.2 |

*Note: The first-measurement cohort required matched PaO₂, hemoglobin, and SpO₂ by design. Reduced denominators for SaO₂, FiO₂, lactate, and APACHE reflect complete-case availability.*

**Table S7. Model denominator audit**

| **Database** | **Analysis** | **First-measurement eligible N** | **Exposure/analysis-variable complete N** | **Final model N** | **Hospital deaths** | **Hospital clusters** |
| --- | --- | --- | --- | --- | --- | --- |
| MIMIC-IV | Hemoglobin P25 (9.10 g/dL) Demographic and diagnosis-derived case-mix Model H: matched hemoglobin burden | 14,052 | 14,050 | 14,050 | 2,823 | — |
| MIMIC-IV | Hemoglobin P25 (9.10 g/dL) Demographic and diagnosis-derived case-mix Model C: CaO₂ burden | 14,052 | 14,050 | 14,050 | 2,823 | — |
| MIMIC-IV | Hemoglobin P25 (9.10 g/dL) Demographic and diagnosis-derived case-mix Model HC: both burdens | 14,052 | 14,050 | 14,050 | 2,823 | — |
| MIMIC-IV | Hemoglobin P25 (9.10 g/dL) Concurrent-treatment/FiO₂ sensitivity Model H: matched hemoglobin burden | 14,052 | 13,676 | 13,676 | 2,695 | — |
| MIMIC-IV | Hemoglobin P25 (9.10 g/dL) Concurrent-treatment/FiO₂ sensitivity Model C: CaO₂ burden | 14,052 | 13,676 | 13,676 | 2,695 | — |
| MIMIC-IV | Hemoglobin P25 (9.10 g/dL) Concurrent-treatment/FiO₂ sensitivity Model HC: both burdens | 14,052 | 13,676 | 13,676 | 2,695 | — |
| MIMIC-IV | Hemoglobin <10 g/dL Demographic and diagnosis-derived case-mix Model H: matched hemoglobin burden | 14,052 | 14,050 | 14,050 | 2,823 | — |
| MIMIC-IV | Hemoglobin <10 g/dL Demographic and diagnosis-derived case-mix Model C: CaO₂ burden | 14,052 | 14,050 | 14,050 | 2,823 | — |
| MIMIC-IV | Hemoglobin <10 g/dL Demographic and diagnosis-derived case-mix Model HC: both burdens | 14,052 | 14,050 | 14,050 | 2,823 | — |
| MIMIC-IV | Hemoglobin <10 g/dL Concurrent-treatment/FiO₂ sensitivity Model H: matched hemoglobin burden | 14,052 | 13,676 | 13,676 | 2,695 | — |
| MIMIC-IV | Hemoglobin <10 g/dL Concurrent-treatment/FiO₂ sensitivity Model C: CaO₂ burden | 14,052 | 13,676 | 13,676 | 2,695 | — |
| MIMIC-IV | Hemoglobin <10 g/dL Concurrent-treatment/FiO₂ sensitivity Model HC: both burdens | 14,052 | 13,676 | 13,676 | 2,695 | — |
| MIMIC-IV | Demographic and diagnosis-derived case-mix | 14,052 | 14,050 | 14,050 | 2,823 | — |
| MIMIC-IV | Concurrent-treatment/FiO₂ sensitivity | 14,052 | 14,050 | 13,676 | 2,695 | — |
| MIMIC-IV | True 24-h landmark | 14,052 | 12,956 | 12,956 | 2,316 | — |
| MIMIC-IV | Fixed-unit and categorical Demographic and diagnosis-derived case-mix | 14,052 | 14,050 | 14,050 | 2,823 | — |
| MIMIC-IV | Fixed-unit and categorical Concurrent-treatment/FiO₂ sensitivity | 14,052 | 13,676 | 13,676 | 2,695 | — |
| MIMIC-IV | Model D: window summaries + one CaO₂ burden | 14,052 | 14,050 | 14,050 | 2,823 | — |
| MIMIC-IV | Model E: window summaries + three component-deficit burdens | 14,052 | 14,050 | 14,050 | 2,823 | — |
| eICU | Hemoglobin P25 (9.10 g/dL) Demographic and diagnosis-derived case-mix Model H: matched hemoglobin burden | 20,802 | 20,780 | 20,780 | 4,051 | 180 |
| eICU | Hemoglobin P25 (9.10 g/dL) Demographic and diagnosis-derived case-mix Model C: CaO₂ burden | 20,802 | 20,780 | 20,780 | 4,051 | 180 |
| eICU | Hemoglobin P25 (9.10 g/dL) Demographic and diagnosis-derived case-mix Model HC: both burdens | 20,802 | 20,780 | 20,780 | 4,051 | 180 |
| eICU | Hemoglobin P25 (9.10 g/dL) Concurrent-treatment/FiO₂ sensitivity Model H: matched hemoglobin burden | 20,802 | 16,091 | 16,091 | 3,244 | 147 |
| eICU | Hemoglobin P25 (9.10 g/dL) Concurrent-treatment/FiO₂ sensitivity Model C: CaO₂ burden | 20,802 | 16,091 | 16,091 | 3,244 | 147 |
| eICU | Hemoglobin P25 (9.10 g/dL) Concurrent-treatment/FiO₂ sensitivity Model HC: both burdens | 20,802 | 16,091 | 16,091 | 3,244 | 147 |
| eICU | Hemoglobin <10 g/dL Demographic and diagnosis-derived case-mix Model H: matched hemoglobin burden | 20,802 | 20,780 | 20,780 | 4,051 | 180 |
| eICU | Hemoglobin <10 g/dL Demographic and diagnosis-derived case-mix Model C: CaO₂ burden | 20,802 | 20,780 | 20,780 | 4,051 | 180 |
| eICU | Hemoglobin <10 g/dL Demographic and diagnosis-derived case-mix Model HC: both burdens | 20,802 | 20,780 | 20,780 | 4,051 | 180 |
| eICU | Hemoglobin <10 g/dL Concurrent-treatment/FiO₂ sensitivity Model H: matched hemoglobin burden | 20,802 | 16,091 | 16,091 | 3,244 | 147 |
| eICU | Hemoglobin <10 g/dL Concurrent-treatment/FiO₂ sensitivity Model C: CaO₂ burden | 20,802 | 16,091 | 16,091 | 3,244 | 147 |
| eICU | Hemoglobin <10 g/dL Concurrent-treatment/FiO₂ sensitivity Model HC: both burdens | 20,802 | 16,091 | 16,091 | 3,244 | 147 |
| eICU | Demographic and diagnosis-derived case-mix | 20,802 | 20,780 | 20,780 | 4,051 | 180 |
| eICU | Base + APACHE | 20,802 | 20,780 | 18,450 | 3,660 | 167 |
| eICU | Concurrent-treatment/FiO₂ sensitivity | 20,802 | 20,780 | 16,091 | 3,244 | 147 |
| eICU | True 24-h landmark | 20,802 | 18,325 | 18,325 | 3,148 | 179 |
| eICU | Fixed-unit and categorical Demographic and diagnosis-derived case-mix | 20,802 | 20,780 | 20,780 | 4,051 | 180 |
| eICU | Fixed-unit and categorical Concurrent-treatment/FiO₂ sensitivity | 20,802 | 16,091 | 16,091 | 3,244 | 147 |
| eICU | Model D: window summaries + one CaO₂ burden | 20,802 | 20,780 | 20,780 | 4,051 | — |
| eICU | Model E: window summaries + three component-deficit burdens | 20,802 | 20,780 | 20,780 | 4,051 | — |

*Note: Eligible N is the first-measurement cohort. Exposure/analysis-variable complete N is the common sample available for the listed comparison; Model N is the final complete-case denominator. Events are hospital deaths, except that landmark rows count deaths after hour 24. A dash indicates that hospital clustering was not applicable to that model.*

**Table S8. Component analysis**

| **Metric** | **MIMIC-IV** | **eICU** |
| --- | --- | --- |
| First matched measurement N | 14,052 | 20,802 |
| MIMIC-locked first-Hb P25, g/dL | 9.100 | 9.100 |
| CaO₂-Hb Pearson r | 0.965 | 0.949 |
| CaO₂-Hb Spearman rho | 0.967 | 0.958 |
| R2 from CaO₂ ~ Hb | 0.932 | 0.901 |
| Dissolved contribution median, % | 2.884 | 2.532 |
| Dissolved contribution Q1, % | 1.586 | 1.728 |
| Dissolved contribution Q3, % | 5.276 | 4.009 |
| Low-CaO₂ N | 3,513 | 4,715 |
| Low-CaO₂ with Hb <9, n | 2,976 | 3,748 |
| Low-CaO₂ with Hb <9, % | 84.714 | 79.491 |
| Low-CaO₂ with Hb <10, n | 3,435 | 4,496 |
| Low-CaO₂ with Hb <10, % | 97.780 | 95.355 |
| SpO₂-preserved low-CaO₂ N | 3,203 | 4,004 |
| Preserved low-CaO₂ with Hb <9, n | 2,831 | 3,436 |
| Preserved low-CaO₂ with Hb <9, % | 88.386 | 85.814 |
| Preserved low-CaO₂ with Hb <10, n | 3,203 | 4,003 |
| Preserved low-CaO₂ with Hb <10, % | 100.000 | 99.975 |
| Burden-pair N | 14,050 | 20,780 |
| CaO₂ burden-matched hemoglobin burden Pearson r | 0.949 | 0.919 |
| CaO₂ burden-matched hemoglobin burden Spearman rho | 0.912 | 0.896 |

*Note: Dissolved contribution is 100 × (0.003 × PaO₂)/CaO₂. R² and percentage content contribution are different quantities.*

**Table S9. Matched hemoglobin-burden and CaO₂-burden models**

| **Database** | **Hb threshold** | **Adjustment** | **Model** | **Term** | **N / events** | **OR (95% CI)** | **5-fold CV AUROC** | **5-fold CV Brier** |
| --- | --- | --- | --- | --- | --- | --- | --- | --- |
| MIMIC-IV | P25 9.10 g/dL | Base | H: covariates + matched hemoglobin burden | Matched hemoglobin burden | 14,050 / 2,823 | 1.21 (1.16–1.26) | 0.742 | 0.139 |
| MIMIC-IV | P25 9.10 g/dL | Base | C: covariates + CaO₂ burden | CaO₂ burden | 14,050 / 2,823 | 1.33 (1.28–1.39) | 0.748 | 0.138 |
| MIMIC-IV | P25 9.10 g/dL | Base | HC: covariates + matched hemoglobin burden + CaO₂ burden | Matched hemoglobin burden | 14,050 / 2,823 | 0.43 (0.37–0.50) | 0.753 | 0.136 |
| MIMIC-IV | P25 9.10 g/dL | Base | HC: covariates + matched hemoglobin burden + CaO₂ burden | CaO₂ burden | 14,050 / 2,823 | 3.01 (2.58–3.50) | 0.753 | 0.136 |
| MIMIC-IV | P25 9.10 g/dL | Concurrent-treatment sensitivity | H: covariates + matched hemoglobin burden | Matched hemoglobin burden | 13,676 / 2,695 | 1.23 (1.18–1.29) | 0.749 | 0.136 |
| MIMIC-IV | P25 9.10 g/dL | Concurrent-treatment sensitivity | C: covariates + CaO₂ burden | CaO₂ burden | 13,676 / 2,695 | 1.36 (1.30–1.42) | 0.754 | 0.134 |
| MIMIC-IV | P25 9.10 g/dL | Concurrent-treatment sensitivity | HC: covariates + matched hemoglobin burden + CaO₂ burden | Matched hemoglobin burden | 13,676 / 2,695 | 0.46 (0.39–0.54) | 0.758 | 0.133 |
| MIMIC-IV | P25 9.10 g/dL | Concurrent-treatment sensitivity | HC: covariates + matched hemoglobin burden + CaO₂ burden | CaO₂ burden | 13,676 / 2,695 | 2.82 (2.41–3.30) | 0.758 | 0.133 |
| MIMIC-IV | Hb <10 g/dL | Base | H: covariates + matched hemoglobin burden | Matched hemoglobin burden | 14,050 / 2,823 | 1.20 (1.15–1.25) | 0.741 | 0.14 |
| MIMIC-IV | Hb <10 g/dL | Base | C: covariates + CaO₂ burden | CaO₂ burden | 14,050 / 2,823 | 1.33 (1.28–1.39) | 0.748 | 0.138 |
| MIMIC-IV | Hb <10 g/dL | Base | HC: covariates + matched hemoglobin burden + CaO₂ burden | Matched hemoglobin burden | 14,050 / 2,823 | 0.48 (0.42–0.55) | 0.755 | 0.136 |
| MIMIC-IV | Hb <10 g/dL | Base | HC: covariates + matched hemoglobin burden + CaO₂ burden | CaO₂ burden | 14,050 / 2,823 | 2.59 (2.28–2.94) | 0.755 | 0.136 |
| MIMIC-IV | Hb <10 g/dL | Concurrent-treatment sensitivity | H: covariates + matched hemoglobin burden | Matched hemoglobin burden | 13,676 / 2,695 | 1.22 (1.16–1.27) | 0.747 | 0.136 |
| MIMIC-IV | Hb <10 g/dL | Concurrent-treatment sensitivity | C: covariates + CaO₂ burden | CaO₂ burden | 13,676 / 2,695 | 1.36 (1.30–1.42) | 0.754 | 0.134 |
| MIMIC-IV | Hb <10 g/dL | Concurrent-treatment sensitivity | HC: covariates + matched hemoglobin burden + CaO₂ burden | Matched hemoglobin burden | 13,676 / 2,695 | 0.50 (0.44–0.57) | 0.76 | 0.133 |
| MIMIC-IV | Hb <10 g/dL | Concurrent-treatment sensitivity | HC: covariates + matched hemoglobin burden + CaO₂ burden | CaO₂ burden | 13,676 / 2,695 | 2.51 (2.20–2.86) | 0.76 | 0.133 |
| eICU | P25 9.10 g/dL | Base | H: covariates + matched hemoglobin burden | Matched hemoglobin burden | 20,780 / 4,051 | 1.15 (1.09–1.20) | 0.667 | 0.147 |
| eICU | P25 9.10 g/dL | Base | C: covariates + CaO₂ burden | CaO₂ burden | 20,780 / 4,051 | 1.29 (1.24–1.35) | 0.677 | 0.145 |
| eICU | P25 9.10 g/dL | Base | HC: covariates + matched hemoglobin burden + CaO₂ burden | Matched hemoglobin burden | 20,780 / 4,051 | 0.51 (0.45–0.57) | 0.685 | 0.143 |
| eICU | P25 9.10 g/dL | Base | HC: covariates + matched hemoglobin burden + CaO₂ burden | CaO₂ burden | 20,780 / 4,051 | 2.42 (2.17–2.70) | 0.685 | 0.143 |
| eICU | P25 9.10 g/dL | Concurrent-treatment sensitivity | H: covariates + matched hemoglobin burden | Matched hemoglobin burden | 16,091 / 3,244 | 1.10 (1.04–1.17) | 0.718 | 0.144 |
| eICU | P25 9.10 g/dL | Concurrent-treatment sensitivity | C: covariates + CaO₂ burden | CaO₂ burden | 16,091 / 3,244 | 1.23 (1.18–1.29) | 0.722 | 0.142 |
| eICU | P25 9.10 g/dL | Concurrent-treatment sensitivity | HC: covariates + matched hemoglobin burden + CaO₂ burden | Matched hemoglobin burden | 16,091 / 3,244 | 0.53 (0.46–0.62) | 0.726 | 0.14 |
| eICU | P25 9.10 g/dL | Concurrent-treatment sensitivity | HC: covariates + matched hemoglobin burden + CaO₂ burden | CaO₂ burden | 16,091 / 3,244 | 2.20 (1.95–2.47) | 0.726 | 0.14 |
| eICU | Hb <10 g/dL | Base | H: covariates + matched hemoglobin burden | Matched hemoglobin burden | 20,780 / 4,051 | 1.11 (1.05–1.16) | 0.664 | 0.147 |
| eICU | Hb <10 g/dL | Base | C: covariates + CaO₂ burden | CaO₂ burden | 20,780 / 4,051 | 1.29 (1.24–1.35) | 0.677 | 0.145 |
| eICU | Hb <10 g/dL | Base | HC: covariates + matched hemoglobin burden + CaO₂ burden | Matched hemoglobin burden | 20,780 / 4,051 | 0.44 (0.40–0.50) | 0.693 | 0.142 |
| eICU | Hb <10 g/dL | Base | HC: covariates + matched hemoglobin burden + CaO₂ burden | CaO₂ burden | 20,780 / 4,051 | 2.65 (2.39–2.94) | 0.693 | 0.142 |
| eICU | Hb <10 g/dL | Concurrent-treatment sensitivity | H: covariates + matched hemoglobin burden | Matched hemoglobin burden | 16,091 / 3,244 | 1.04 (0.98–1.11) | 0.717 | 0.144 |
| eICU | Hb <10 g/dL | Concurrent-treatment sensitivity | C: covariates + CaO₂ burden | CaO₂ burden | 16,091 / 3,244 | 1.23 (1.18–1.29) | 0.722 | 0.142 |
| eICU | Hb <10 g/dL | Concurrent-treatment sensitivity | HC: covariates + matched hemoglobin burden + CaO₂ burden | Matched hemoglobin burden | 16,091 / 3,244 | 0.42 (0.37–0.48) | 0.734 | 0.138 |
| eICU | Hb <10 g/dL | Concurrent-treatment sensitivity | HC: covariates + matched hemoglobin burden + CaO₂ burden | CaO₂ burden | 16,091 / 3,244 | 2.67 (2.37–2.99) | 0.734 | 0.138 |

*Note: All H, C, and HC comparisons use identical samples and adjustment sets. Joint HC coefficients are not interpreted independently because of collinearity.*

**Table S10. Landmark, fixed-unit, and categorical results**

| **Section** | **Database** | **Adjustment** | **Analysis / level** | **N / events** | **OR (95% CI)** | **Burden SD** | **SE approach** |
| --- | --- | --- | --- | --- | --- | --- | --- |
| True 24-h landmark | MIMIC-IV | Base | Alive and ICU-observable at 24 h | 12,956 / 2,316 | 1.26 (1.21–1.32) |  | model-based |
| True 24-h landmark | eICU | Base | Alive and ICU-observable at 24 h | 18,325 / 3,148 | 1.19 (1.14–1.25) |  | hospital-cluster robust |
| Per fixed 0.5 mL O2/dL burden increase | MIMIC-IV | Base | Continuous | 14,050 / 2,823 | 1.37 (1.31–1.43) | 0.456 | model-based |
| Burden categories | MIMIC-IV | Base | Zero burden (reference) | 14,050 / 2,823 | 1.00 (1.00–1.00) | 0.456 | model-based |
| Burden categories | MIMIC-IV | Base | Positive T1 | 14,050 / 2,823 | 0.85 (0.73–0.99) | 0.456 | model-based |
| Burden categories | MIMIC-IV | Base | Positive T2 | 14,050 / 2,823 | 1.46 (1.27–1.67) | 0.456 | model-based |
| Burden categories | MIMIC-IV | Base | Positive T3 | 14,050 / 2,823 | 2.23 (1.96–2.53) | 0.456 | model-based |
| Per fixed 0.5 mL O2/dL burden increase | MIMIC-IV | Concurrent-treatment sensitivity | Continuous | 13,676 / 2,695 | 1.40 (1.33–1.47) | 0.456 | model-based |
| Burden categories | MIMIC-IV | Concurrent-treatment sensitivity | Zero burden (reference) | 13,676 / 2,695 | 1.00 (1.00–1.00) | 0.456 | model-based |
| Burden categories | MIMIC-IV | Concurrent-treatment sensitivity | Positive T1 | 13,676 / 2,695 | 0.83 (0.71–0.97) | 0.456 | model-based |
| Burden categories | MIMIC-IV | Concurrent-treatment sensitivity | Positive T2 | 13,676 / 2,695 | 1.56 (1.35–1.80) | 0.456 | model-based |
| Burden categories | MIMIC-IV | Concurrent-treatment sensitivity | Positive T3 | 13,676 / 2,695 | 2.32 (2.01–2.67) | 0.456 | model-based |
| Per fixed 0.5 mL O2/dL burden increase | eICU | Base | Continuous | 20,780 / 4,051 | 1.38 (1.31–1.45) | 0.4 | hospital-cluster robust |
| Burden categories | eICU | Base | Zero burden (reference) | 20,780 / 4,051 | 1.00 (1.00–1.00) | 0.4 | hospital-cluster robust |
| Burden categories | eICU | Base | Positive T1 | 20,780 / 4,051 | 1.03 (0.92–1.15) | 0.4 | hospital-cluster robust |
| Burden categories | eICU | Base | Positive T2 | 20,780 / 4,051 | 1.23 (1.10–1.37) | 0.4 | hospital-cluster robust |
| Burden categories | eICU | Base | Positive T3 | 20,780 / 4,051 | 2.10 (1.82–2.43) | 0.4 | hospital-cluster robust |
| Per fixed 0.5 mL O2/dL burden increase | eICU | Concurrent-treatment sensitivity | Continuous | 16,091 / 3,244 | 1.29 (1.22–1.36) | 0.4 | hospital-cluster robust |
| Burden categories | eICU | Concurrent-treatment sensitivity | Zero burden (reference) | 16,091 / 3,244 | 1.00 (1.00–1.00) | 0.4 | hospital-cluster robust |
| Burden categories | eICU | Concurrent-treatment sensitivity | Positive T1 | 16,091 / 3,244 | 0.91 (0.81–1.04) | 0.4 | hospital-cluster robust |
| Burden categories | eICU | Concurrent-treatment sensitivity | Positive T2 | 16,091 / 3,244 | 1.12 (0.98–1.27) | 0.4 | hospital-cluster robust |
| Burden categories | eICU | Concurrent-treatment sensitivity | Positive T3 | 16,091 / 3,244 | 1.74 (1.50–2.03) | 0.4 | hospital-cluster robust |

**Table S11. Cross-validated exploratory model performance**

| **Database** | **Comparison** | **Threshold / contrast** | **Adjustment set** | **Model** | **N / events** | **AIC** | **BIC** | **5-fold CV AUROC** | **5-fold CV Brier** | **Condition number** |
| --- | --- | --- | --- | --- | --- | --- | --- | --- | --- | --- |
| MIMIC-IV | H/C/HC | P25 9.10 g/dL | Demographic and diagnosis-derived case-mix | H: covariates + matched hemoglobin burden | 14,050 / 2,823 | 12346.0 | 12474.3 | 0.742 | 0.139 | 21.4 |
| MIMIC-IV | H/C/HC | P25 9.10 g/dL | Demographic and diagnosis-derived case-mix | C: covariates + CaO₂ burden | 14,050 / 2,823 | 12239.9 | 12368.2 | 0.748 | 0.138 | 21.4 |
| MIMIC-IV | H/C/HC | P25 9.10 g/dL | Demographic and diagnosis-derived case-mix | HC: covariates + matched hemoglobin burden + CaO₂ burden | 14,050 / 2,823 | 12097.4 | 12233.3 | 0.753 | 0.136 | 21.4 |
| MIMIC-IV | H/C/HC | P25 9.10 g/dL | Concurrent treatment/FiO₂ sensitivity | H: covariates + matched hemoglobin burden | 13,676 / 2,695 | 11774.7 | 11932.7 | 0.749 | 0.136 | 23.4 |
| MIMIC-IV | H/C/HC | P25 9.10 g/dL | Concurrent treatment/FiO₂ sensitivity | C: covariates + CaO₂ burden | 13,676 / 2,695 | 11676.1 | 11834.1 | 0.754 | 0.134 | 23.4 |
| MIMIC-IV | H/C/HC | P25 9.10 g/dL | Concurrent treatment/FiO₂ sensitivity | HC: covariates + matched hemoglobin burden + CaO₂ burden | 13,676 / 2,695 | 11568.0 | 11733.5 | 0.758 | 0.133 | 23.5 |
| MIMIC-IV | H/C/HC | Hb <10 g/dL | Demographic and diagnosis-derived case-mix | H: covariates + matched hemoglobin burden | 14,050 / 2,823 | 12359.2 | 12487.5 | 0.741 | 0.14 | 21.4 |
| MIMIC-IV | H/C/HC | Hb <10 g/dL | Demographic and diagnosis-derived case-mix | C: covariates + CaO₂ burden | 14,050 / 2,823 | 12239.9 | 12368.2 | 0.748 | 0.138 | 21.4 |
| MIMIC-IV | H/C/HC | Hb <10 g/dL | Demographic and diagnosis-derived case-mix | HC: covariates + matched hemoglobin burden + CaO₂ burden | 14,050 / 2,823 | 12105.0 | 12241.0 | 0.755 | 0.136 | 21.4 |
| MIMIC-IV | H/C/HC | Hb <10 g/dL | Concurrent treatment/FiO₂ sensitivity | H: covariates + matched hemoglobin burden | 13,676 / 2,695 | 11790.0 | 11948.0 | 0.747 | 0.136 | 23.4 |
| MIMIC-IV | H/C/HC | Hb <10 g/dL | Concurrent treatment/FiO₂ sensitivity | C: covariates + CaO₂ burden | 13,676 / 2,695 | 11676.1 | 11834.1 | 0.754 | 0.134 | 23.4 |
| MIMIC-IV | H/C/HC | Hb <10 g/dL | Concurrent treatment/FiO₂ sensitivity | HC: covariates + matched hemoglobin burden + CaO₂ burden | 13,676 / 2,695 | 11566.9 | 11732.4 | 0.76 | 0.133 | 23.5 |
| eICU | H/C/HC | P25 9.10 g/dL | Demographic and diagnosis-derived case-mix | H: covariates + matched hemoglobin burden | 20,780 / 4,051 | 19303.8 | 19454.7 | 0.667 | 0.147 | 75.9 |
| eICU | H/C/HC | P25 9.10 g/dL | Demographic and diagnosis-derived case-mix | C: covariates + CaO₂ burden | 20,780 / 4,051 | 19115.7 | 19266.6 | 0.677 | 0.145 | 75.9 |
| eICU | H/C/HC | P25 9.10 g/dL | Demographic and diagnosis-derived case-mix | HC: covariates + matched hemoglobin burden + CaO₂ burden | 20,780 / 4,051 | 18867.7 | 19026.6 | 0.685 | 0.143 | 76.0 |
| eICU | H/C/HC | P25 9.10 g/dL | Concurrent treatment/FiO₂ sensitivity | H: covariates + matched hemoglobin burden | 16,091 / 3,244 | 14534.0 | 14710.8 | 0.718 | 0.144 | 75.1 |
| eICU | H/C/HC | P25 9.10 g/dL | Concurrent treatment/FiO₂ sensitivity | C: covariates + CaO₂ burden | 16,091 / 3,244 | 14436.4 | 14613.1 | 0.722 | 0.142 | 75.1 |
| eICU | H/C/HC | P25 9.10 g/dL | Concurrent treatment/FiO₂ sensitivity | HC: covariates + matched hemoglobin burden + CaO₂ burden | 16,091 / 3,244 | 14283.1 | 14467.5 | 0.726 | 0.14 | 75.1 |
| eICU | H/C/HC | Hb <10 g/dL | Demographic and diagnosis-derived case-mix | H: covariates + matched hemoglobin burden | 20,780 / 4,051 | 19335.4 | 19486.3 | 0.664 | 0.147 | 75.9 |
| eICU | H/C/HC | Hb <10 g/dL | Demographic and diagnosis-derived case-mix | C: covariates + CaO₂ burden | 20,780 / 4,051 | 19115.7 | 19266.6 | 0.677 | 0.145 | 75.9 |
| eICU | H/C/HC | Hb <10 g/dL | Demographic and diagnosis-derived case-mix | HC: covariates + matched hemoglobin burden + CaO₂ burden | 20,780 / 4,051 | 18746.7 | 18905.5 | 0.693 | 0.142 | 76.0 |
| eICU | H/C/HC | Hb <10 g/dL | Concurrent treatment/FiO₂ sensitivity | H: covariates + matched hemoglobin burden | 16,091 / 3,244 | 14553.1 | 14729.9 | 0.717 | 0.144 | 75.1 |
| eICU | H/C/HC | Hb <10 g/dL | Concurrent treatment/FiO₂ sensitivity | C: covariates + CaO₂ burden | 16,091 / 3,244 | 14436.4 | 14613.1 | 0.722 | 0.142 | 75.1 |
| eICU | H/C/HC | Hb <10 g/dL | Concurrent treatment/FiO₂ sensitivity | HC: covariates + matched hemoglobin burden + CaO₂ burden | 16,091 / 3,244 | 14131.8 | 14316.2 | 0.734 | 0.138 | 75.1 |
| MIMIC-IV | D versus E | Not applicable | Original parsimony comparison | D: window summaries + one CaO₂ burden | 14,050 / 2,823 | 12215.9 | 12344.2 | 0.748 | 0.138 | 25.9 |
| MIMIC-IV | D versus E | Not applicable | Original parsimony comparison | E: window summaries + three component-deficit burdens | 14,050 / 2,823 | 12201.4 | 12344.8 | 0.748 | 0.138 | 27.3 |
| MIMIC-IV | D versus E | D minus E | Original parsimony comparison | D minus E | 14,050 / 2,823 | 14.5 | -0.6 | -0.0 | 0.0 |  |
| eICU | D versus E | Not applicable | Original parsimony comparison | D: window summaries + one CaO₂ burden | 20,780 / 4,051 | 18776.1 | 18927.0 | 0.683 | 0.141 | 94.4 |
| eICU | D versus E | Not applicable | Original parsimony comparison | E: window summaries + three component-deficit burdens | 20,780 / 4,051 | 18734.8 | 18901.6 | 0.684 | 0.141 | 99.5 |
| eICU | D versus E | D minus E | Original parsimony comparison | D minus E | 20,780 / 4,051 | 41.3 | 25.5 | -0.001 | 0.0 |  |

*Note: AUROC and Brier scores use deterministic stratified five-fold cross-validation. Differences are exploratory and were not evaluated for clinical decision usefulness.*

**Table S12. Measurement coverage and observed-time sensitivity**

| **Model ID** | **Database** | **Analysis definition** | **Cohort source** | **Exposure** | **N / deaths** | **Exposure mean (SD)** | **Coverage, h, median [Q1, Q3]** | **OR (95% CI)** | **Adjustment set** | **SE / clusters** | **Reference exposure SD** |
| --- | --- | --- | --- | --- | --- | --- | --- | --- | --- | --- | --- |
| MP-A | MIMIC-IV | Primary fixed-24-h burden with measurement coverage adjustment | Primary burden cohort | 24-hour low-CaO₂ burden, per database SD | 14,050 / 2,823 | 0.201 (0.456) | 12.00 [7.93, 17.58] | 1.31 (1.25–1.36) | Demographics + diagnosis-derived case-mix + measurement coverage covariates | model-based | 0.456 |
| MP-B-SD | MIMIC-IV | Observed-time mean deficit restricted to coverage ≥6 h | Primary burden cohort with coverage ≥6 h | Observed-time mean deficit, coverage ≥6 h, per database SD | 13,534 / 2,714 | 0.349 (0.761) | 12.00 [8.84, 17.82] | 1.35 (1.29–1.40) | Demographics + diagnosis-derived case-mix | model-based | 0.761 |
| MP-B-0.5 | MIMIC-IV | Observed-time mean deficit restricted to coverage ≥6 h | Primary burden cohort with coverage ≥6 h | Observed-time mean deficit, coverage ≥6 h, per 0.5 mL O₂/dL | 13,534 / 2,714 | 0.349 (0.761) | 12.00 [8.84, 17.82] | 1.22 (1.18–1.25) | Demographics + diagnosis-derived case-mix | model-based | 0.761 |
| MP-C-SD | MIMIC-IV | Observed-time mean deficit restricted to coverage ≥12 h | Primary burden cohort with coverage ≥12 h | Observed-time mean deficit, coverage ≥12 h, per database SD | 7,473 / 1,707 | 0.391 (0.742) | 17.22 [13.92, 20.95] | 1.29 (1.22–1.37) | Demographics + diagnosis-derived case-mix | model-based | 0.742 |
| MP-C-0.5 | MIMIC-IV | Observed-time mean deficit restricted to coverage ≥12 h | Primary burden cohort with coverage ≥12 h | Observed-time mean deficit, coverage ≥12 h, per 0.5 mL O₂/dL | 7,473 / 1,707 | 0.391 (0.742) | 17.22 [13.92, 20.95] | 1.19 (1.15–1.23) | Demographics + diagnosis-derived case-mix | model-based | 0.742 |
| MP-A | eICU | Primary fixed-24-h burden with measurement coverage adjustment | Primary burden cohort | 24-hour low-CaO₂ burden, per database SD | 20,780 / 4,051 | 0.176 (0.400) | 10.08 [6.00, 13.62] | 1.29 (1.24–1.33) | Demographics + diagnosis-derived case-mix + measurement coverage covariates | hospital-cluster robust; 180 hospitals | 0.400 |
| MP-B-SD | eICU | Observed-time mean deficit restricted to coverage ≥6 h | Primary burden cohort with coverage ≥6 h | Observed-time mean deficit, coverage ≥6 h, per database SD | 19,399 / 3,780 | 0.375 (0.827) | 10.67 [6.00, 14.10] | 1.33 (1.28–1.37) | Demographics + diagnosis-derived case-mix | hospital-cluster robust; 179 hospitals | 0.827 |
| MP-B-0.5 | eICU | Observed-time mean deficit restricted to coverage ≥6 h | Primary burden cohort with coverage ≥6 h | Observed-time mean deficit, coverage ≥6 h, per 0.5 mL O₂/dL | 19,399 / 3,780 | 0.375 (0.827) | 10.67 [6.00, 14.10] | 1.19 (1.16–1.21) | Demographics + diagnosis-derived case-mix | hospital-cluster robust; 179 hospitals | 0.827 |
| MP-C-SD | eICU | Observed-time mean deficit restricted to coverage ≥12 h | Primary burden cohort with coverage ≥12 h | Observed-time mean deficit, coverage ≥12 h, per database SD | 7,952 / 1,668 | 0.419 (0.759) | 15.27 [12.43, 18.13] | 1.24 (1.17–1.31) | Demographics + diagnosis-derived case-mix | hospital-cluster robust; 157 hospitals | 0.759 |
| MP-C-0.5 | eICU | Observed-time mean deficit restricted to coverage ≥12 h | Primary burden cohort with coverage ≥12 h | Observed-time mean deficit, coverage ≥12 h, per 0.5 mL O₂/dL | 7,952 / 1,668 | 0.419 (0.759) | 15.27 [12.43, 18.13] | 1.15 (1.11–1.20) | Demographics + diagnosis-derived case-mix | hospital-cluster robust; 157 hospitals | 0.759 |

*Note: Observed-time mean deficit divides deficit area by measured coverage and is restricted to the stated minimum coverage. Values are rounded for display; confidence intervals, rather than duplicate P values, are shown for inference.*

**Table S13. eICU repeated-patient audit and one-observation-per-patient sensitivity**

| **Section** | **Analysis / metric** | **Value or N / deaths** | **OR (95% CI)** | **Hospital clusters** | **Notes** |
| --- | --- | --- | --- | --- | --- |
| Primary-cohort audit | Included eICU unit stays | 20,802 |  |  | Earliest hospitaldischargeyear, then lowest patientunitstayid; exact within-year chronology unavailable |
| Primary-cohort audit | Unique patients | 20,090 |  |  |  |
| Primary-cohort audit | Patients represented more than once | 630 (3.14%) |  |  |  |
| Primary-cohort audit | Unit stays belonging to repeated patients | 1,342 |  |  |  |
| Primary-cohort audit | Maximum included stays per patient | 6 |  |  |  |
| Repeat-count distribution | 1 included stay(s) per patient | 19,460 patients |  |  |  |
| Repeat-count distribution | 2 included stay(s) per patient | 571 patients |  |  |  |
| Repeat-count distribution | 3 included stay(s) per patient | 42 patients |  |  |  |
| Repeat-count distribution | 4 included stay(s) per patient | 13 patients |  |  |  |
| Repeat-count distribution | 5 included stay(s) per patient | 2 patients |  |  |  |
| Repeat-count distribution | 6 included stay(s) per patient | 2 patients |  |  |  |
| One-observation-per-patient sensitivity | One observation per eICU patient, diagnosis-derived case-mix model | 20,068 / 3,957 | 1.30 (1.25–1.36) |  | Demographics + diagnosis-derived case-mix |
| One-observation-per-patient sensitivity | One observation per eICU patient, APACHE sensitivity | 17,816 / 3,576 | 1.14 (1.10–1.19) |  | Demographics + diagnosis-derived case-mix |
| One-observation-per-patient sensitivity | One observation per eICU patient, alive and still in index ICU at 24 h | 17,673 / 3,073 | 1.20 (1.15–1.26) |  | Demographics + diagnosis-derived case-mix |

**Table S14. Locked positive-burden cut points and categorical models**

| **Section** | **Database** | **Analysis** | **Category / numeric range** | **N / deaths** | **OR (95% CI)** | **Adjustment** | **SE / clusters** |
| --- | --- | --- | --- | --- | --- | --- | --- |
| Locked positive-burden cut points | MIMIC-IV | Locked primary burden cohort, positive burden only | T1 upper 0.16521375; T2 upper 0.59175987 mL O₂/dL | Positive burden n=5,113 |  | Not applicable | Not applicable |
| Locked positive-burden cut points | eICU | Locked primary burden cohort, positive burden only | T1 upper 0.21392882; T2 upper 0.61046495 mL O₂/dL | Positive burden n=6,705 |  | Not applicable | Not applicable |
| Categorical model | MIMIC-IV | Base diagnosis-derived case-mix | Zero burden: 0 | 8,937 / 1,464 | Reference | Demographics + diagnosis-derived case-mix | model-based |
| Categorical model | MIMIC-IV | Base diagnosis-derived case-mix | Positive T1: >0 to ≤0.16521375 | 1,705 / 262 | 0.85 (0.73–0.99) | Demographics + diagnosis-derived case-mix | model-based |
| Categorical model | MIMIC-IV | Base diagnosis-derived case-mix | Positive T2: >0.16521375 to ≤0.591759872 | 1,704 / 430 | 1.46 (1.27–1.67) | Demographics + diagnosis-derived case-mix | model-based |
| Categorical model | MIMIC-IV | Base diagnosis-derived case-mix | Positive T3: >0.591759872 | 1,704 / 667 | 2.23 (1.96–2.53) | Demographics + diagnosis-derived case-mix | model-based |
| Categorical model | MIMIC-IV | Concurrent treatment/FiO₂ sensitivity | Zero burden: 0 | 8,710 / 1,407 | Reference | Demographics + diagnosis-derived case-mix + concurrent treatment/FiO₂ | model-based |
| Categorical model | MIMIC-IV | Concurrent treatment/FiO₂ sensitivity | Positive T1: >0 to ≤0.16521375 | 1,682 / 253 | 0.85 (0.72–0.99) | Demographics + diagnosis-derived case-mix + concurrent treatment/FiO₂ | model-based |
| Categorical model | MIMIC-IV | Concurrent treatment/FiO₂ sensitivity | Positive T2: >0.16521375 to ≤0.591759872 | 1,656 / 409 | 1.54 (1.34–1.78) | Demographics + diagnosis-derived case-mix + concurrent treatment/FiO₂ | model-based |
| Categorical model | MIMIC-IV | Concurrent treatment/FiO₂ sensitivity | Positive T3: >0.591759872 | 1,628 / 626 | 2.35 (2.04–2.71) | Demographics + diagnosis-derived case-mix + concurrent treatment/FiO₂ | model-based |
| Categorical model | MIMIC-IV | Landmark: alive and still in index ICU at 24 h | Zero burden: 0 | 8,227 / 1,229 | Reference | Demographics + diagnosis-derived case-mix | model-based |
| Categorical model | MIMIC-IV | Landmark: alive and still in index ICU at 24 h | Positive T1: >0 to ≤0.16521375 | 1,601 / 217 | 0.82 (0.70–0.97) | Demographics + diagnosis-derived case-mix | model-based |
| Categorical model | MIMIC-IV | Landmark: alive and still in index ICU at 24 h | Positive T2: >0.16521375 to ≤0.591759872 | 1,587 / 353 | 1.35 (1.17–1.57) | Demographics + diagnosis-derived case-mix | model-based |
| Categorical model | MIMIC-IV | Landmark: alive and still in index ICU at 24 h | Positive T3: >0.591759872 | 1,541 / 517 | 1.86 (1.61–2.13) | Demographics + diagnosis-derived case-mix | model-based |
| Categorical model | eICU | Base diagnosis-derived case-mix | Zero burden: 0 | 14,075 / 2,342 | Reference | Demographics + diagnosis-derived case-mix | hospital-cluster robust; 180 hospitals |
| Categorical model | eICU | Base diagnosis-derived case-mix | Positive T1: >0 to ≤0.213928819 | 2,235 / 429 | 1.03 (0.92–1.15) | Demographics + diagnosis-derived case-mix | hospital-cluster robust; 180 hospitals |
| Categorical model | eICU | Base diagnosis-derived case-mix | Positive T2: >0.213928819 to ≤0.610464951 | 2,235 / 507 | 1.23 (1.10–1.37) | Demographics + diagnosis-derived case-mix | hospital-cluster robust; 180 hospitals |
| Categorical model | eICU | Base diagnosis-derived case-mix | Positive T3: >0.610464951 | 2,235 / 773 | 2.10 (1.82–2.43) | Demographics + diagnosis-derived case-mix | hospital-cluster robust; 180 hospitals |
| Categorical model | eICU | Concurrent treatment/FiO₂ sensitivity | Zero burden: 0 | 10,757 / 1,874 | Reference | Demographics + diagnosis-derived case-mix + concurrent treatment/FiO₂ | hospital-cluster robust; 147 hospitals |
| Categorical model | eICU | Concurrent treatment/FiO₂ sensitivity | Positive T1: >0 to ≤0.213928819 | 1,732 / 337 | 0.91 (0.80–1.04) | Demographics + diagnosis-derived case-mix + concurrent treatment/FiO₂ | hospital-cluster robust; 147 hospitals |
| Categorical model | eICU | Concurrent treatment/FiO₂ sensitivity | Positive T2: >0.213928819 to ≤0.610464951 | 1,772 / 409 | 1.11 (0.98–1.27) | Demographics + diagnosis-derived case-mix + concurrent treatment/FiO₂ | hospital-cluster robust; 147 hospitals |
| Categorical model | eICU | Concurrent treatment/FiO₂ sensitivity | Positive T3: >0.610464951 | 1,830 / 624 | 1.72 (1.48–2.01) | Demographics + diagnosis-derived case-mix + concurrent treatment/FiO₂ | hospital-cluster robust; 147 hospitals |
| Categorical model | eICU | Landmark: alive and still in index ICU at 24 h | Zero burden: 0 | 12,433 / 1,905 | Reference | Demographics + diagnosis-derived case-mix | hospital-cluster robust; 179 hospitals |
| Categorical model | eICU | Landmark: alive and still in index ICU at 24 h | Positive T1: >0 to ≤0.213928819 | 2,016 / 347 | 1.00 (0.89–1.13) | Demographics + diagnosis-derived case-mix | hospital-cluster robust; 179 hospitals |
| Categorical model | eICU | Landmark: alive and still in index ICU at 24 h | Positive T2: >0.213928819 to ≤0.610464951 | 1,985 / 383 | 1.10 (0.97–1.25) | Demographics + diagnosis-derived case-mix | hospital-cluster robust; 179 hospitals |
| Categorical model | eICU | Landmark: alive and still in index ICU at 24 h | Positive T3: >0.610464951 | 1,891 / 513 | 1.62 (1.38–1.90) | Demographics + diagnosis-derived case-mix | hospital-cluster robust; 179 hospitals |

*Note: Positive-burden cut points were calculated once in each primary cohort and reused. Zero burden is the reference category.*

**Table S15. Collinearity diagnostics for joint hemoglobin and CaO₂ burden models**

| **Database** | **Adjustment set** | **Hemoglobin threshold** | **N / events** | **Term** | **Burden Pearson r** | **VIF** | **Tolerance** | **Condition number** |
| --- | --- | --- | --- | --- | --- | --- | --- | --- |
| MIMIC-IV | Demographic and diagnosis-derived case-mix | MIMIC-IV first hemoglobin P25 = 9.10 g/dL | 14,050 / 2,823 | Matched hemoglobin burden | 0.949 | 10.25 | 0.098 | 21.4 |
| MIMIC-IV | Demographic and diagnosis-derived case-mix | MIMIC-IV first hemoglobin P25 = 9.10 g/dL | 14,050 / 2,823 | CaO₂ burden | 0.949 | 10.40 | 0.096 | 21.4 |
| MIMIC-IV | Demographic and diagnosis-derived case-mix | Hb <10 g/dL | 14,050 / 2,823 | Matched hemoglobin burden | 0.924 | 7.22 | 0.138 | 21.4 |
| MIMIC-IV | Demographic and diagnosis-derived case-mix | Hb <10 g/dL | 14,050 / 2,823 | CaO₂ burden | 0.924 | 7.13 | 0.140 | 21.4 |
| MIMIC-IV | Concurrent treatment/FiO₂ sensitivity | MIMIC-IV first hemoglobin P25 = 9.10 g/dL | 13,676 / 2,695 | Matched hemoglobin burden | 0.948 | 10.50 | 0.095 | 23.5 |
| MIMIC-IV | Concurrent treatment/FiO₂ sensitivity | MIMIC-IV first hemoglobin P25 = 9.10 g/dL | 13,676 / 2,695 | CaO₂ burden | 0.948 | 10.50 | 0.095 | 23.5 |
| MIMIC-IV | Concurrent treatment/FiO₂ sensitivity | Hb <10 g/dL | 13,676 / 2,695 | Matched hemoglobin burden | 0.924 | 7.55 | 0.132 | 23.5 |
| MIMIC-IV | Concurrent treatment/FiO₂ sensitivity | Hb <10 g/dL | 13,676 / 2,695 | CaO₂ burden | 0.924 | 7.17 | 0.139 | 23.5 |
| eICU | Demographic and diagnosis-derived case-mix | MIMIC-IV first hemoglobin P25 = 9.10 g/dL | 20,780 / 4,051 | Matched hemoglobin burden | 0.919 | 6.51 | 0.154 | 76.0 |
| eICU | Demographic and diagnosis-derived case-mix | MIMIC-IV first hemoglobin P25 = 9.10 g/dL | 20,780 / 4,051 | CaO₂ burden | 0.919 | 6.53 | 0.153 | 76.0 |
| eICU | Demographic and diagnosis-derived case-mix | Hb <10 g/dL | 20,780 / 4,051 | Matched hemoglobin burden | 0.897 | 5.18 | 0.193 | 76.0 |
| eICU | Demographic and diagnosis-derived case-mix | Hb <10 g/dL | 20,780 / 4,051 | CaO₂ burden | 0.897 | 5.18 | 0.193 | 76.0 |
| eICU | Concurrent treatment/FiO₂ sensitivity | MIMIC-IV first hemoglobin P25 = 9.10 g/dL | 16,091 / 3,244 | Matched hemoglobin burden | 0.922 | 6.91 | 0.145 | 75.1 |
| eICU | Concurrent treatment/FiO₂ sensitivity | MIMIC-IV first hemoglobin P25 = 9.10 g/dL | 16,091 / 3,244 | CaO₂ burden | 0.922 | 6.91 | 0.145 | 75.1 |
| eICU | Concurrent treatment/FiO₂ sensitivity | Hb <10 g/dL | 16,091 / 3,244 | Matched hemoglobin burden | 0.900 | 5.42 | 0.185 | 75.1 |
| eICU | Concurrent treatment/FiO₂ sensitivity | Hb <10 g/dL | 16,091 / 3,244 | CaO₂ burden | 0.900 | 5.39 | 0.185 | 75.1 |

*Note: VIFs were calculated from the same design matrices used in the joint Hb–CaO₂ models. Values above 5 indicate substantial overlap between the two burden measures; joint coefficients should not be interpreted as independent effects.*

**Table S16. Post hoc SpO₂-stratified component and model-performance analysis**

| **Database** | **First SpO₂ stratum** | **Anchor N** | **Model N/deaths** | **Pearson r** | **Spearman rho** | **R²** | **H AUROC** | **C AUROC** | **ΔAUROC** | **H Brier** | **C Brier** | **ΔBrier** |
| --- | --- | --- | --- | --- | --- | --- | --- | --- | --- | --- | --- | --- |
| MIMIC-IV | SpO₂ <90% | 401 | 399/208 | 0.887 | 0.888 | 0.787 | 0.633 | 0.678 | 0.045 | 0.239 | 0.227 | -0.012 |
| MIMIC-IV | SpO₂ 90-94% | 1,370 | 1,370/474 | 0.995 | 0.995 | 0.990 | 0.722 | 0.729 | 0.007 | 0.198 | 0.195 | -0.003 |
| MIMIC-IV | SpO₂ ≥95% | 12,281 | 12,281/2,141 | 0.987 | 0.986 | 0.973 | 0.742 | 0.744 | 0.002 | 0.126 | 0.126 | -0.001 |
| eICU | SpO₂ <90% | 1,040 | 1,022/541 | 0.826 | 0.853 | 0.682 | 0.608 | 0.651 | 0.044 | 0.242 | 0.233 | -0.009 |
| eICU | SpO₂ 90-94% | 1,944 | 1,944/533 | 0.996 | 0.996 | 0.992 | 0.682 | 0.687 | 0.005 | 0.183 | 0.181 | -0.001 |
| eICU | SpO₂ ≥95% | 17,818 | 17,814/2,977 | 0.989 | 0.988 | 0.978 | 0.664 | 0.667 | 0.002 | 0.131 | 0.131 | 0.000 |

*Note: Post hoc analyses requested by the Associate Editor. Strata use the first matched SpO₂. H is the demographic and diagnosis-derived case-mix model plus matched hemoglobin burden; C replaces hemoglobin burden with CaO₂ burden. H and C use identical samples. AUROC and Brier values use deterministic stratified five-fold cross-validation; deltas are C minus H.*

**Table S17. Cross-validated calibration of matched hemoglobin and CaO₂ burden models**

| **Database** | **Model** | **N/deaths** | **Observed mortality** | **Mean predicted** | **O/E** | **Calibration-in-the-large** | **Joint intercept** | **Calibration slope** | **AUROC** | **Brier** |
| --- | --- | --- | --- | --- | --- | --- | --- | --- | --- | --- |
| MIMIC-IV | H: case mix + Hb burden | 14,050/2,823 | 20.09% | 20.11% | 0.999 | -0.001 | -0.022 | 0.983 | 0.742 | 0.139 |
| MIMIC-IV | C: case mix + CaO₂ burden | 14,050/2,823 | 20.09% | 20.10% | 0.999 | -0.001 | -0.021 | 0.983 | 0.748 | 0.138 |
| eICU | H: case mix + Hb burden | 20,780/4,051 | 19.49% | 19.49% | 1.000 | 0.000 | -0.024 | 0.982 | 0.667 | 0.147 |
| eICU | C: case mix + CaO₂ burden | 20,780/4,051 | 19.49% | 19.49% | 1.000 | 0.000 | -0.021 | 0.984 | 0.677 | 0.145 |

*Note: Calibration uses deterministic stratified five-fold out-of-fold predictions from the same paired samples as the H-versus-C comparison. Calibration-in-the-large is estimated with the prediction logit as an offset; the joint intercept and slope come from regressing outcome on the out-of-fold prediction logit. O/E is observed divided by expected deaths.*
